# Global, high-resolution, reduced-complexity air quality modeling for PM2.5 using InMAP (Intervention Model for Air Pollution)

**DOI:** 10.1371/journal.pone.0268714

**Published:** 2022-05-25

**Authors:** Sumil K. Thakrar, Christopher W. Tessum, Joshua S. Apte, Srinidhi Balasubramanian, Dylan B. Millet, Spyros N. Pandis, Julian D. Marshall, Jason D. Hill

**Affiliations:** 1 Department of Bioproducts & Biosystems Engineering, University of Minnesota, St Paul, Minnesota, United States of America; 2 Department of Applied Economics, University of Minnesota, St Paul, Minnesota, United States of America; 3 Department of Civil and Environmental Engineering, University of Illinois at Urbana−Champaign, Urbana, Illinois, United States of America; 4 Department of Civil and Environmental Engineering, University of California, Berkeley, Berkeley, California, United States of America; 5 School of Public Health, University of California, Berkeley, California, United States of America; 6 Department of Soil, Water, and Climate, University of Minnesota, St Paul, Minnesota, United States of America; 7 Department of Chemical Engineering, Carnegie Mellon University, Pittsburgh, Pennsylvania, United States of America; 8 Department of Chemical Engineering, University of Patras, Patras, Greece; 9 Department of Civil and Environmental Engineering, University of Washington, Seattle, Washington, United States of America; Texas A&M University, UNITED STATES

## Abstract

Each year, millions of premature deaths worldwide are caused by exposure to outdoor air pollution, especially fine particulate matter (PM_2.5_). Designing policies to reduce these deaths relies on air quality modeling for estimating changes in PM_2.5_ concentrations from many scenarios at high spatial resolution. However, air quality modeling typically has substantial requirements for computation and expertise, which limits policy design, especially in countries where most PM_2.5_-related deaths occur. Lower requirement reduced-complexity models exist but are generally unavailable worldwide. Here, we adapt InMAP, a reduced-complexity model originally developed for the United States, to simulate annual-average primary and secondary PM_2.5_ concentrations across a global-through-urban spatial domain: “Global InMAP”. Global InMAP uses a variable resolution grid, with horizontal grid cell widths ranging from 500 km in remote locations to 4km in urban locations. We evaluate Global InMAP performance against both measurements and a state-of-the-science chemical transport model, GEOS-Chem. Against measurements, InMAP predicts total PM_2.5_ concentrations with a normalized mean error of 62%, compared to 41% for GEOS-Chem. For the emission scenarios considered, Global InMAP reproduced GEOS-Chem pollutant concentrations with a normalized mean bias of 59%–121%, which is sufficient for initial policy assessment and scoping. Global InMAP can be run on a desktop computer; simulations here took 2.6–8.4 hours. This work presents a global, open-source, reduced-complexity air quality model to facilitate policy assessment worldwide, providing a screening tool for reducing air pollution-related deaths where they occur most.

## Introduction

Exposure to outdoor air pollution is the largest environmental health risk factor worldwide, associated with millions of excess deaths each year [1,2]. The deaths are mostly attributable to fine particulate matter (PM_2.5_), which can either be emitted directly, or can form indirectly from precursor pollutants that are emitted from a wide variety of biogenic and anthropogenic emission sources, including transportation, agriculture, and electricity generation [3,4]. Designing strategies to reduce mortality relies on understanding how specific emission sources affect ambient PM_2.5_ concentrations, and thereby, human health, across a range of possible technology or policy scenarios.

InMAP [5] (Intervention Model for Air Pollution) is a reduced-complexity, open-source air quality model that has been used to inform strategies to reduce PM_2.5_-related mortality from specific emission sources. For example, InMAP has been used to estimate fine-scale pollution impacts across distances [6], measures of pollution inequity across racial-ethnic and socioeconomic groups [7], the health impacts of specific sectors under different policy scenarios [8], and the impacts of individual activities such as freight [9], electricity generation [10], and maize production [11]. However, as with other widely used reduced-complexity air quality models such as EASIUR [12], AP2 [13], and COBRA [14], InMAP previously has only been configured and evaluated for the United States, a country with just 4% of the world’s population and 2% of the world’s air quality-related deaths [2,4].

Chemical transport models (CTMs) are employed for estimating the effects of emission sources on pollutant concentrations and health impacts and are considered state-of-the-science for air quality modeling. However, they require substantial time, expertise, and computational resources (*e*.*g*., several computation days per simulation month), limiting the use cases and therefore the extent to which they can inform multidimensional policy decisions [5,15], especially when modelling dozens or hundreds of policy scenarios at high spatial resolution. Although GEOS-Chem is one of the most widely used CTMs, 60% of deaths from outdoor air pollution occur in countries where there are no known users or institutions using GEOS-Chem [16,17]. Thus, researchers and practitioners would benefit from additional models and tools beyond CTMs to investigate air pollution and emission control strategies. Such tools would be useful even though the uncertainty may be higher than with a CTM. For example, because damages per tonne emitted vary by orders of magnitude across space [6], for many analyses an uncertainty of a factor of 2 or 3, or higher (e.g., an order of magnitude estimate), can provide scientifically relevant results that can usefully inform policy decisions.

Some global air quality models are available with a lower operational difficulty or computational intensity than CTMs, including TM5-FASST [18], source-receptor relationships built from GEOS-Chem adjoint [19], and EMEP [20]. Compared to the existing global air quality models with lower operational difficulty than CTMs, InMAP has a unique combination of higher spatial resolution, ease of use, and low computational costs. A recent notable effort [21] to build a monthly life cycle assessment model for PM_2.5_ has not yet been tested against measurements or compared with results from a CTM. A diversity of independently evaluated reduced-complexity models will increase their applicability and the robustness of policy assessments worldwide [22].

Here, we developed and configured InMAP for use on a global spatial domain (“Global InMAP”). We ran a year-long, global CTM simulation using GEOS-Chem [23], and used its outputs to globally parameterize the chemistry, physics, and meteorology of InMAP. We then ran InMAP on global emission inventories to predict total PM_2.5_ concentrations and changes in concentrations from three specific scenarios of emission changes. We compared the results to a global dataset of ground observations, and to PM_2.5_ concentrations and changes in concentrations predicted by GEOS-Chem. Lastly, we compared Global InMAP to the United States versions of InMAP for two emission scenarios.

## Materials and methods

The InMAP model, described in Tessum *et al*. [5], estimates annual-average concentrations of fine particulate matter (PM2.5), including both primary (*i*.*e*., directly emitted) and secondary (*i*.*e*., formed in the atmosphere) components, to guide research and policy. As with other reduced-complexity models, InMAP is designed to be faster and easier to use than CTMs, and will typically have lower accuracy and precision than CTMs as a tradeoff for greater speed and ease of use.

InMAP explicitly tracks secondary PM_2.5_ contributions from particulate ammonium (pNH4), particulate sulfate (pSO4), particulate nitrate (pNO3), and secondary organic aerosol (SOA), from emissions of PM2.5 precursors (sulfur oxides (SOx), nitrogen oxides (NOx), ammonia (NH3), and non-methane volatile organic compounds (NMVOCs)). InMAP estimates pollutant concentrations by approximating the steady-state solution to a set of differential equations governing pollutant emissions, reaction, advection, diffusion, and removal. It solves the equations by discretizing over space and time, using a variable resolution grid, and spatially varying parameterizations that simplify the reaction, advection, and removal terms in the equations. Whereas CTMs simulate chemistry and physics (*e*.*g*., reaction, advection, removal) using first principles and mechanistic or empirical representations for specific processes, InMAP simulates chemistry and physics using simplified representations that are parameterized by the outputs of a CTM simulation.

InMAP as configured over the United States (“US InMAP”) was parameterized using outputs from WRF-Chem [24,25]. However, WRF-Chem is not commonly used for global simulations. Instead, InMAP was parameterized here using outputs from GEOS-Chem [23], a global CTM. The full list of equations used in InMAP is given in Tessum *et al*. [5] Details of the model configuration, GEOS-Chem simulation inputs, global emission inventories, and performance evaluation are provided below.

### Global InMAP computational grid

As with previous InMAP configurations for the US [5–11], the horizontal resolution of the Global InMAP computational grid varies across space and is higher in places with larger population or population density. Here, we used 2020 projected population data at 0.01° resolution [26] to create the computational grid. We employed a population density threshold of 5.5 × 10^8^ deg^-2^ and a population threshold of 100,000. Beginning with a 5° × 4° global grid, for any grid cell, if either threshold was exceeded, then the model subdivided it into smaller cells, and iterated the process until either the thresholds were not exceeded or the smallest cell size was reached.

The resulting computational grid (S1 Fig) has ~2.3 million grid cells (ground-level: 273,739 grid cells), whose horizontal resolution at ground-level ranges from 5° × 4° (which corresponds to ~500 km length at the equator) in remote locations to 0.04° × 0.03° (~4 km length at the equator) in urban locations. The spatial domain encompasses the vast majority of the Earth’s surface: latitudes from -87.0° to +81.0° and longitudes from -178.0° to +172.0°. Global InMAP does not track pollution across the poles or antimeridian [27]. The resulting grid covers all but ~5 million people (< 0.1% of the total global population) in parts of New Zealand and other islands in the Pacific Ocean. The population-weighted average grid-cell size is 590 km^2^ (for comparison, ~39,000 km^2^ for GEOS-Chem). The resulting pre-processed gridded input data file is ~1.2 GB and is provided in a freely available dataset (doi:10.5281/zenodo.4641947).

### GEOS-Chem simulation

Chemical and physical atmospheric parameters used in Global InMAP, such as annual-average gas/particle-phase partitioning coefficients, were derived from the outputs of an annual GEOS-Chem “Classic” (version 11-01f) simulation (2016-01-01 until 2017-01-01), with meteorology provided by MERRA-2 [28]. The GEOS-Chem outputs were used in the same way as the corresponding WRF-Chem variables were used for US InMAP (see Tessum *et al*. [5]). The full list of GEOS-Chem variables used in Global InMAP, and descriptions of how they are used, are in Table A in S1 Appendix.

The GEOS-Chem model code and configuration were derived from a simulation performed by Hammer *et al*. [29], where the chemical mechanism included complex secondary organic aerosol (SOA) formation with semi-volatile primary organic aerosol [30,31]. We used the standard horizontal spatial resolution for global simulations in GEOS-Chem, 2° × 2.5°, (~ 220 km × 275 km at the equator) with 47 vertical levels, following the configuration described in Hammer *et al*. [29].

GEOS-Chem also allows for higher resolution grids nested within a larger domain [32]. Again following Hammer *et al*. [29], we ran GEOS-Chem nested grid simulations over the same time period (year 2016) for Asia, Europe, and North America, at 0.5° × 0.625° resolution, which covers 75% of the world’s population. First, boundary conditions for the nested grid simulations were recorded every 180 minutes of simulation time, at 2° × 2.5° resolution, during the global simulation. In our application, emergent properties extracted for use in Global InMAP, such as the annual-average temperature and wind velocity vectors, are only specified up to this coarse resolution. However, Global InMAP can still be used on a higher resolution (variable) grid, and the resolution of the emission inventory is also not limited by the resolution of the GEOS-Chem output.

### Emission inputs

To estimate concentrations of total PM_2.5_ and speciated components using Global InMAP, we compiled a global emission inventory of NH_3_, primary PM_2.5_, NO_x_, SO_x_, and NMVOC. For consistency, we chose the same emission inventories as those used in the GEOS-Chem simulation, but, where possible, processed to a higher spatial resolution as described below. Total annual emissions fluxes for the emission inventories used in the Global InMAP simulation are given in Table 1.

**Table 1:** PM_2.5_ and precursor emissions inputs into GEOS-Chem and Global InMAP.

Pollutant	GEOS-Chem (Tg yr^-1^)	Global InMAP (Tg yr^-1^)	Global InMAP data sources	Maximum resolution
*Anthropogenic*				
PM_2.5_	24.45	32.93	EDGAR, NEI, CAC, MEIC	0.25° × 0.25°
NH_3_	51.52	47.39	EDGAR, CAC, NEI, MIX, MEIC	0.25° × 0.25°
SO_x_	84.33	84.33	EDGAR, BRAVO, EMEP, NEI, CAC, MIX, MEIC, Lu *et al*.	2° × 2.5°
NO_x_	64.85	76.28	EDGAR, BRAVO, EMEP, NEI, CAC, MIX, MEIC, AEIC	0.25° × 0.25°
NMVOC	- ^b^	58.15	EDGAR	0.1° × 0.1°
*Natural*				
PM_2.5_	244.53	244.53	DEAD, GEOS-Chem diagnostics	2° × 2.5°
NH_3_	17.38	15.97	GEIA	0.25° × 0.25°
SO_x_	28.32	0.42^a^	Ge *et al*., GEOS-Chem diagnostics	2° × 2.5°
NO_x_	28.02	16.60^a^	Hudman *et al*., GEOS-Chem diagnostics	2° × 2.5°
NMVOC	- ^b^	553.14	MEGAN, GEOS-Chem diagnostics	2° × 2.5°
*Biomass burning*				
PM_2.5_	35.30	35.30	GFED-4	0.25° × 0.25°
NH_3_	4.24	4.24	GFED-4	0.25° × 0.25°
SO_x_	2.25	2.25	GFED-4	0.25° × 0.25°
NO_x_	20.28	20.28	GFED-4	0.25° × 0.25°
NMVOC	- ^b^	5.10	RETRO	0.5° × 0.5°

^a^Only NO_x_ and SO_x_ emissions in the lowest vertical layer were used in Global InMAP, yet the majority of natural NO_x_ and SO_x_ emissions are emitted from lightning and volcanoes at higher levels.

^b^Not all NMVOC emissions from GEOS-Chem simulation are reported.

Where possible, the total emission inventories used for the Global InMAP simulation were compiled using the standalone version of HEMCO [33], using the same configuration as used in the GEOS-Chem simulation except at 0.25° × 0.25° horizontal resolution.

Differences in grid resolutions, time steps, and environmental fields can result in small differences when the same emission inventories are processed. HEMCO standalone provides both high resolution emissions and consistency with the GEOS-Chem simulation but cannot be used for some emission inventories that require detailed chemical or meteorological inputs. For those, we instead saved out emissions (“diagnostics”) from the GEOS-Chem simulation, gridded at 2° × 2.5°, and used these in the global InMAP simulation.

Table 1 gives the total annual emissions for Global InMAP inputs, and the data source for each group of emissions used. Global and regional emission inventories used for anthropogenic sources of PM_2.5_ and precursors include: EDGAR [34] v.4.3.2, the National Emissions Inventory (NEI) 2011 for the United States, BRAVO [35] (Big Bend Regional Aerosol and Visibility Observational study) for Mexico, the Criteria Air Contaminant (CAC) emission inventory for Canada, EMEP [36] for Europe, MIX [37] v1.1 for Asia, MEIC [38] v1.2 for China, Lu *et al*. [39] for SO_x_ emissions in China and India, AEIC [40] for aircraft emissions, PARANOX [41] for ship emissions, and RETRO [42] for biofuel emissions. Biomass burning emissions are from the RETRO [42] and GFED-4 [43] emission inventories. Natural emission inventories used here include Ge *et al*. [44] for volcanic emissions, Hudman *et al*. [45] for soil NO_x_, MEGAN [46] for biogenic emissions, and DEAD [47] for dust emissions.

Only a subset of NMVOC emissions is likely to form SOA [48,49]. For Global InMAP anthropogenic emissions, we included isoprene, monoterpenes, benzene, toluene, xylenes, trimethylbenzenes, alkanes with more than 4 carbon atoms, and other aromatics, from the EDGAR [34] v4.3.2 emission inventory. For biogenic emissions, we included limonene, isoprene, alpha-pinene, beta-pinene, sabinene, carene, and monoterpenes from the global GEOS-Chem simulation. For biomass burning, we include benzene, toluene, xylenes, alkenes with more than 3 carbon atoms, and alkanes with more than 4 carbon atoms, from the RETRO biomass burning emission inventory [42].

Although Global InMAP has the functionality to include vertically elevated emissions, there is a lack of global information on emission heights for many sources [34]. HEMCO processed emissions were thus derived at the lowest vertical layer, except for aircraft emissions, lightning NO_x_, and volcanic SO_x_. For simplicity in configuring the Global InMAP emissions, here we only used the emissions from these sources in the lowest vertical layer, which excluded 8% of global NO_x_ emissions and 16% of global SO_x_ emissions.

PM_2.5_ concentrations are not directly tracked in GEOS-Chem, but rather are calculated from its underlying components that are grouped in such a way as to facilitate chemical and atmospheric modeling. For example, dust is grouped by several size classes that do not perfectly map onto PM_2.5_. HEMCO and GEOS-Chem diagnostic outputs also typically report emissions in these groups, requiring conversion for use in Global InMAP. Here, we did so in accordance with the standard GEOS-Chem recommendations (see Table A in S1 Appendix for the PM_2.5_ equation used). Following Hammer *et al*. [29] and Li *et al*. [50], irreversible aqueous formation of SOA from isoprene was included in total PM_2.5_ mass, whereas reversible formation was excluded.

InMAP data inputs for pollutant removal through deposition likewise required modification for Global InMAP simulations. Specifically, Global InMAP requires land cover data to calculate dry deposition rates for gases and particles in each ground-level grid cell. For the United States, InMAP used land cover data from the United States Geological Survey National Land Cover Database [51]. For Global InMAP, we instead used the Olson 2001 Land Use Map at 0.025° × 0.025° resolution [52], which is also used in GEOS-Chem.

### Comparison with other air quality models and measurements

Using the global emission inventories described in the previous section, we generated Global InMAP results and compared them against (1) measurements of total and speciated PM_2.5_ concentrations; (2) another model (GEOS-Chem) for three perturbation scenarios, wherein we modified global emissions from a specific sector and predicted the resulting concentration changes; and (3) an earlier version of the same model (US InMAP), for United States electricity and transportation emissions.

First, we evaluated Global InMAP predictions of PM_2.5_ (total and speciated) against annual-average ground-level measurements, as is commonly done for air quality models [53,54]. To this end, we compiled and vetted a global measurement dataset for total and speciated PM_2.5_ (see Text A and Table B in S1 Appendix for additional details). We reported metrics commonly used for evaluating model performance: normalized mean error and bias (NME and NMB), the squared linear correlation coefficient, R^2^, and the slope of the best-fit line, S (see Text B in S1 Appendix for the equations) [55]. Using this approach, model-measurement comparisons were generated for Global InMAP and (separately) for the GEOS-Chem simulation (described above). Model criteria are often reported to provide context for model-measurement comparison results [55,56]. Here, we report model criteria published by Emery *et al*. [55] (see S2 Text).

Second, we simulated the effects of three emissions perturbations with Global InMAP and GEOS-Chem simulations and compared their predicted pollutant concentration increments. The perturbations chosen were: (i) a 100% increase (4.9 Tg) in global SO_2_ emissions from power generation for 2 months (2016-01-01 until 2016-03-01); (ii) a 100% increase (7.5 Tg) in global NH_3_ emissions from agricultural soils for 3 months (2016-01-01 until 2016-04-01); (iii) a 100% increase (1.4 Tg) in global NO_x_ emissions from road transport for 1 month (2016-01-01 until 2016-02-01). All emissions changes were from the EDGAR emissions database (v.4.2, 0.1° × 0.1° resolution) as described above. For each of the scenarios chosen, we ran global, annual 2° × 2.5° GEOS-Chem simulations similar to those described above, with the change in emissions implemented using a uniform temporal profile over the timescale of the perturbation. As InMAP is an “intervention” model (designed to model changes in emissions directly), for Global InMAP we ran the changes in emissions from the EDGAR emission inventories at native resolution.

Lastly, because InMAP has already been configured and evaluated over the contiguous United States, we performed two simulations for United States emission changes using Global InMAP and US InMAP. To this end, we compiled emission inventories over the United States using the National Emissions Inventory (NEI) 2014v.1, processed exactly as in Thakrar *et al*. [8]. We investigated two sources of PM_2.5_ and precursor emissions: coal-powered electricity generation (NEI Source Classification Code: 10100212) and gasoline passenger vehicles (NEI Source Classification Code: 2201210080).

## Results

### Computational requirements

The annual, global simulations described above (system: 98 processors on 1 node of a supercomputing cluster; 36 GB memory) required 8.4 hours for Global InMAP (2.3 million grid cells) and 100 hours for GEOS-Chem (2° × 2.5° grid resolution, 0.6 million grid cells). The perturbation simulations, when run on the same system, took 2.6–4.4 hours.

Other GEOS-Chem simulations require comparably high resources [57]. The variable resolution InMAP grid allows for much higher spatial resolution over areas with high population density than is possible with the GEOS-Chem uniform grid, while only requiring 8% of the computational time.

### Model-to-measurement comparisons

**Fig 1:**
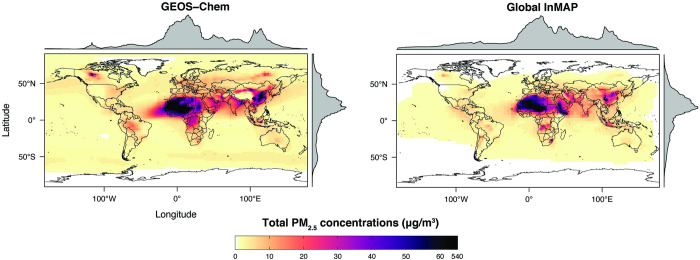
Annual-average ground-level total PM_2.5_ concentrations from the Global InMAP and GEOS-Chem simulations for year 2016.

**Fig 2:**
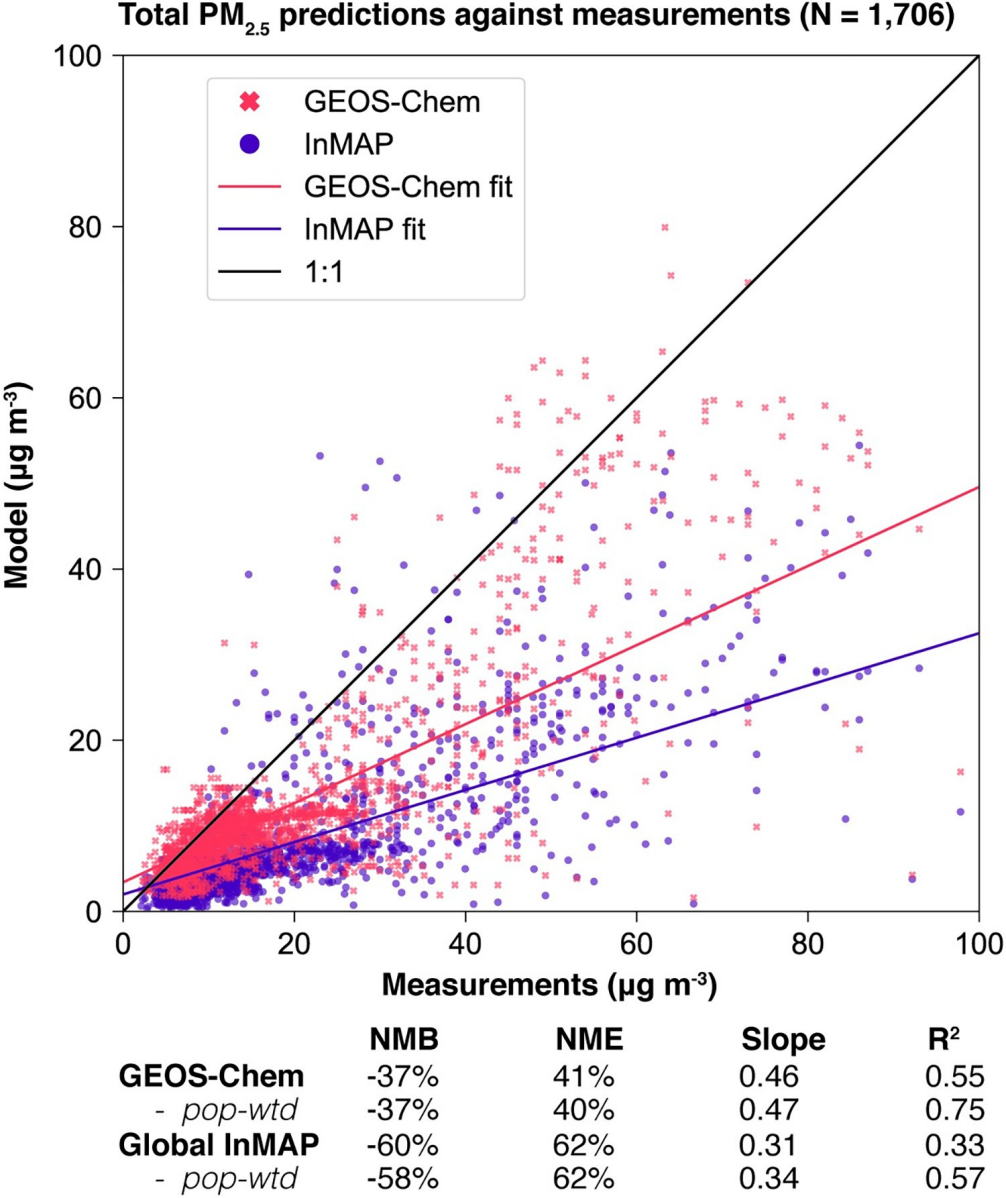
Annual-average total PM2.5 concentrations from the Global InMAP and GEOS-Chem simulations against measurements. Only values ≤100 μg m^-3^ are plotted here, excluding 25 (1.5%) model-measurement pairs (full figure shown in S2 Fig).

The Global InMAP simulation using total emissions predicted total PM_2.5_ concentrations against measurements globally with NMB = –60%; NME = 62%; and R^2^ = 0.33 (see Figs 1 and 2, S2). For comparison, the GEOS Chem simulation predicted total PM_2.5_ concentrations against measurements with NMB = –37%; NME = 41%; and R^2^ = 0.55. As with the GEOS-Chem simulation, the performance of the Global InMAP simulation varied by region (see S3–S9 Figs; Table C in S1 Appendix). By region, the Global InMAP simulation was generally most accurate in Oceania (NMB: -49%; R^2^: 0.82; see S7 Fig), North America (NMB: -45%; R^2^: 0.92; see S6 Fig), and Europe (NMB: -64%; R^2^: 0.30; see S5 Fig), and least accurate in South America (NMB: -76%; R^2^: 0.05; see S8 Fig). The inaccurate prediction in South America may have arisen from discrepancies in emission inventories [58]. Across many heavily polluted regions in Asia, the Global InMAP simulation predicted much lower PM_2.5_ concentrations than are measured (difference: > 30 μg m^-3^) (S5 Fig), in particular across the Indo-Gangetic Plain. The underprediction may have arisen because of potentially low emissions inputs, *e*.*g*. from industrial and agricultural NH_3_ emissions [59] or missing NMVOC species from biomass burning [60]. The Global InMAP simulation may have underpredicted pollution from episodic events, such as biomass burning in the Indo-Gangetic Plain, because Global InMAP assumes that emissions occur at an annual-average rate, whereas PM_2.5_ attributable to biomass burning in that region is largest during times of year with lower than average dispersion conditions [61,62]. Furthermore, the chemistry that is included in Global InMAP may not be sufficiently complex to predict PM_2.5_ with high accuracy in certain polluted areas [63].

**Fig 3:**
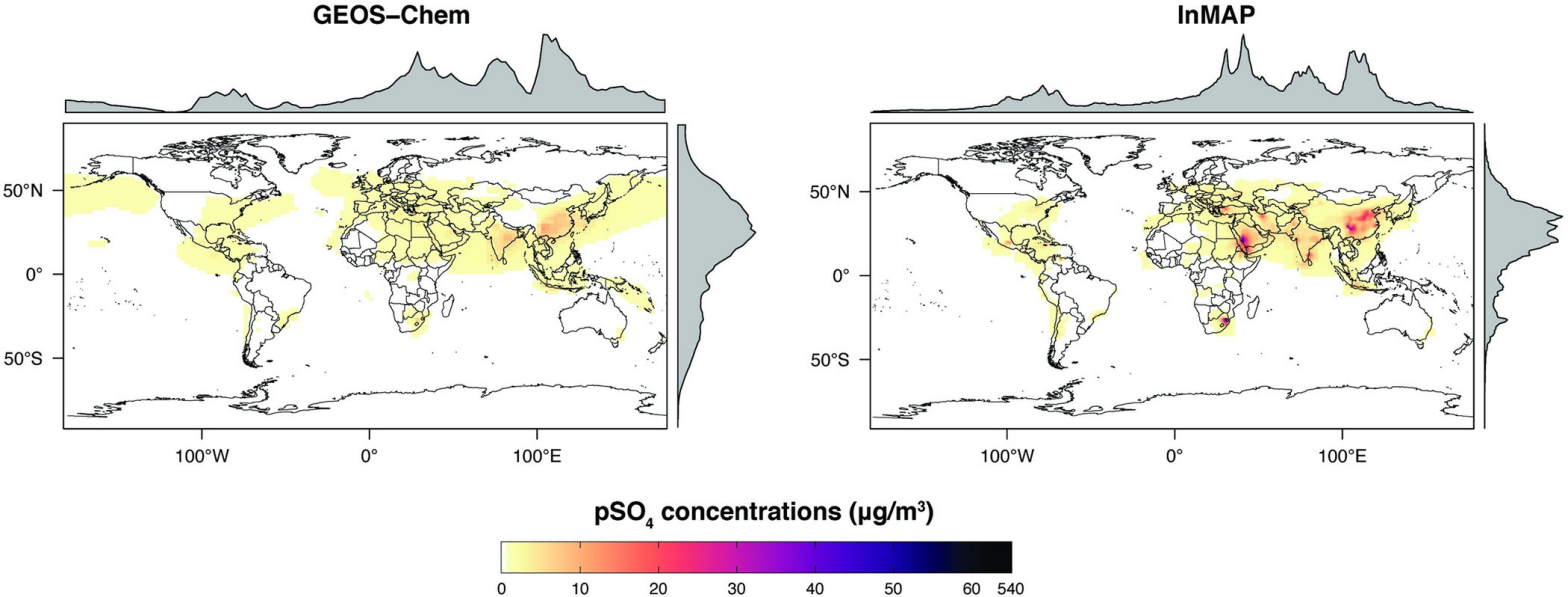
Global InMAP and GEOS-Chem annual-average ground-level pSO_4_ concentrations.

**Fig 4:**
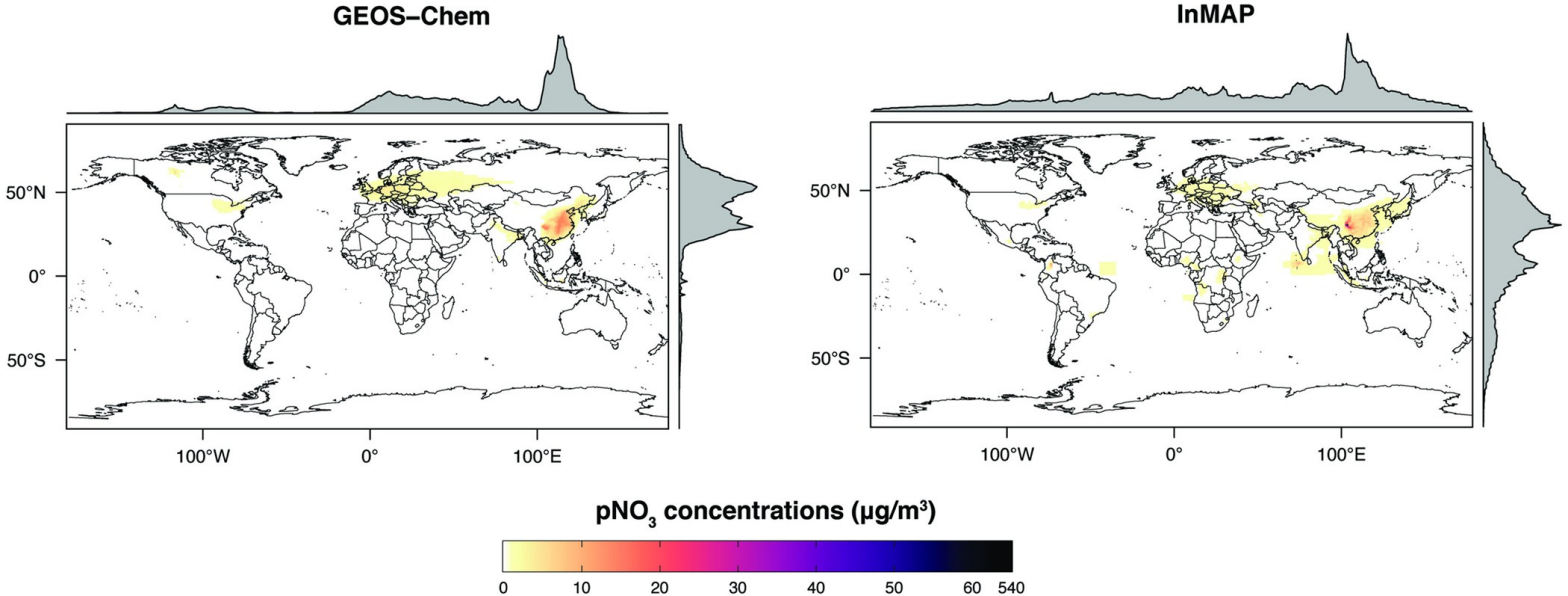
Global InMAP and GEOS-Chem annual-average ground-level pNO_3_ concentrations.

**Fig 5:**
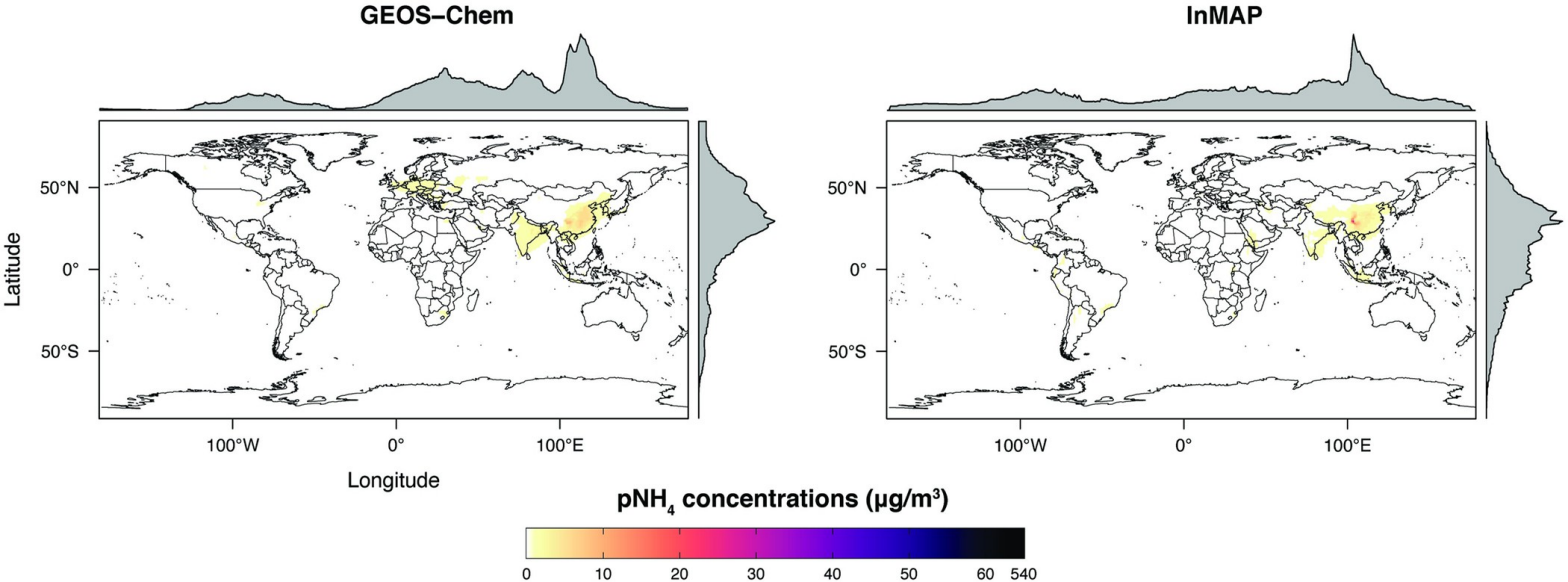
Global InMAP and GEOS-Chem annual-average ground-level pNH_4_ concentrations.

**Fig 6:**
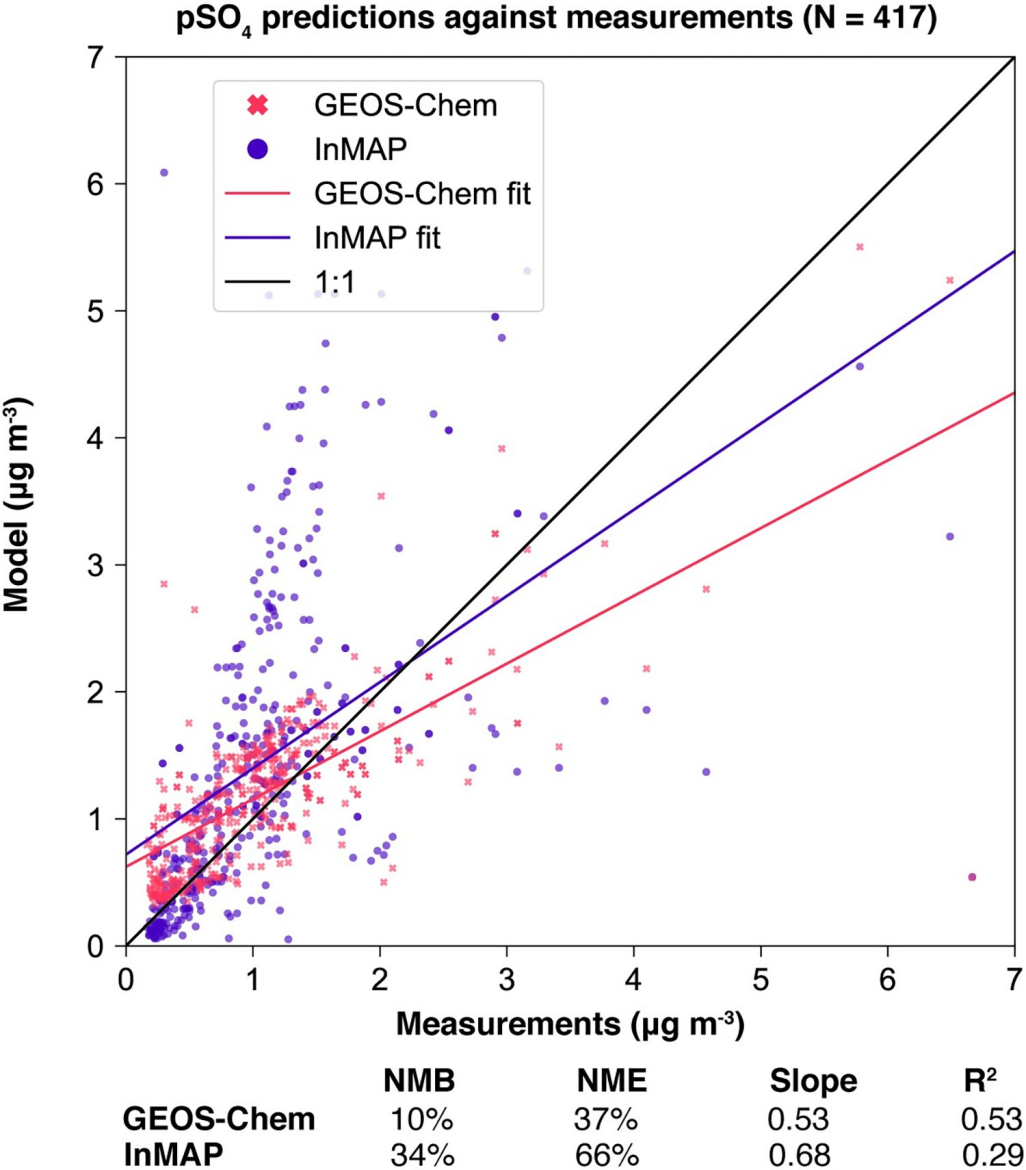
Global InMAP and GEOS-Chem annual-average pSO_4_ concentrations against measurements.

**Fig 7:**
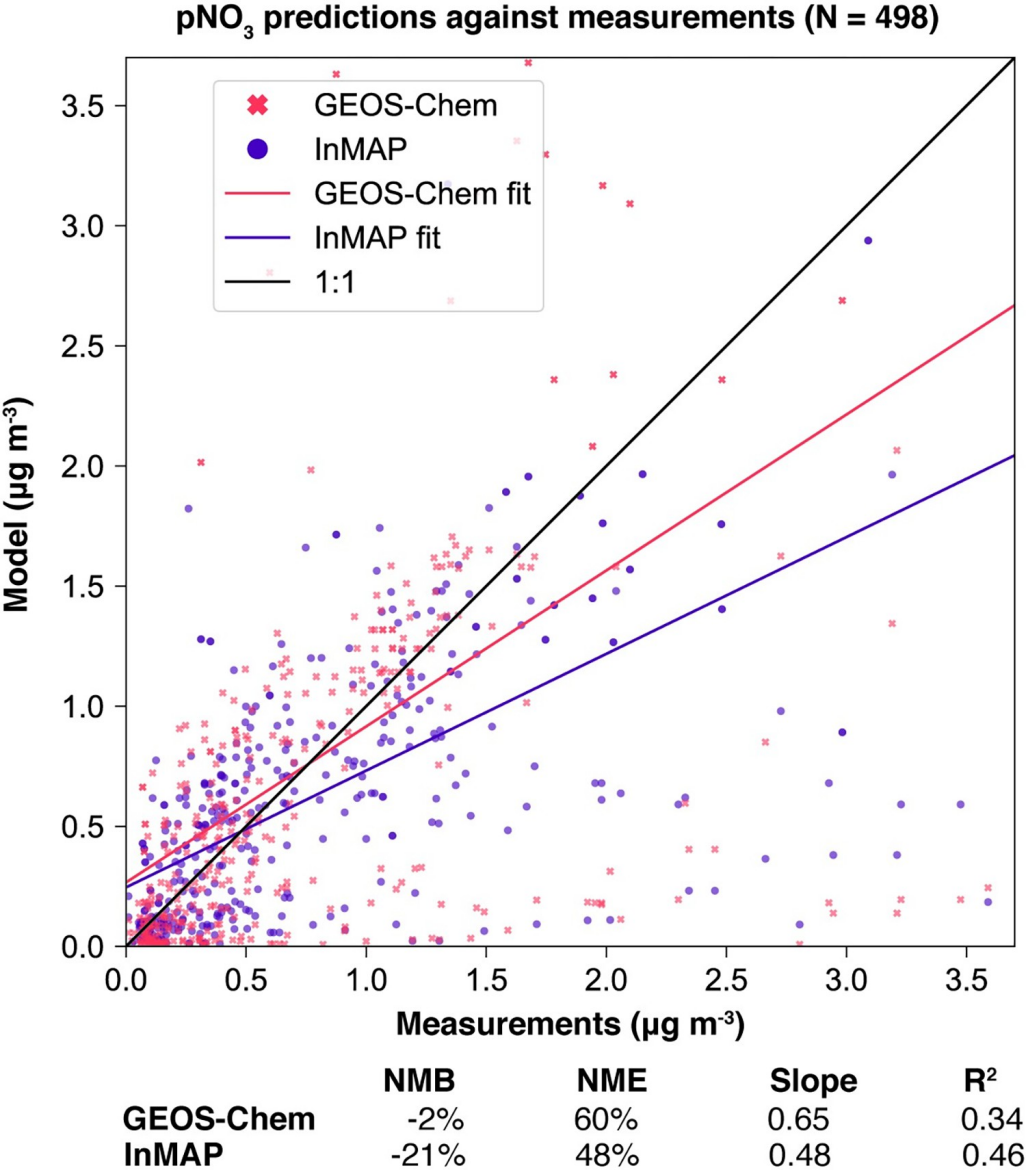
Global InMAP and GEOS-Chem annual-average pNO_3_ concentrations against measurements.

**Fig 8:**
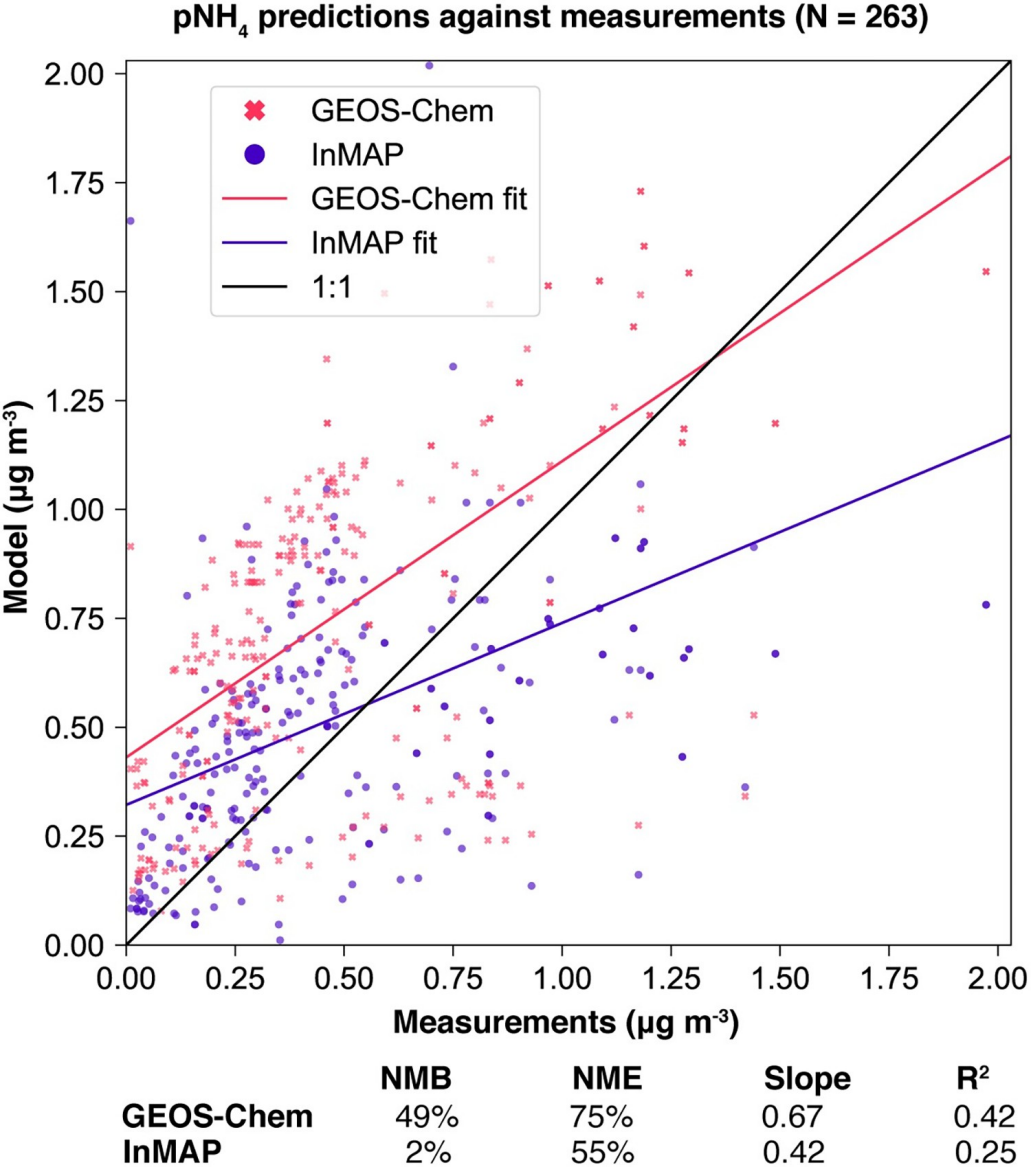
Global InMAP and GEOS-Chem annual-average pNH_4_ concentrations against measurements.

**Fig 9:**
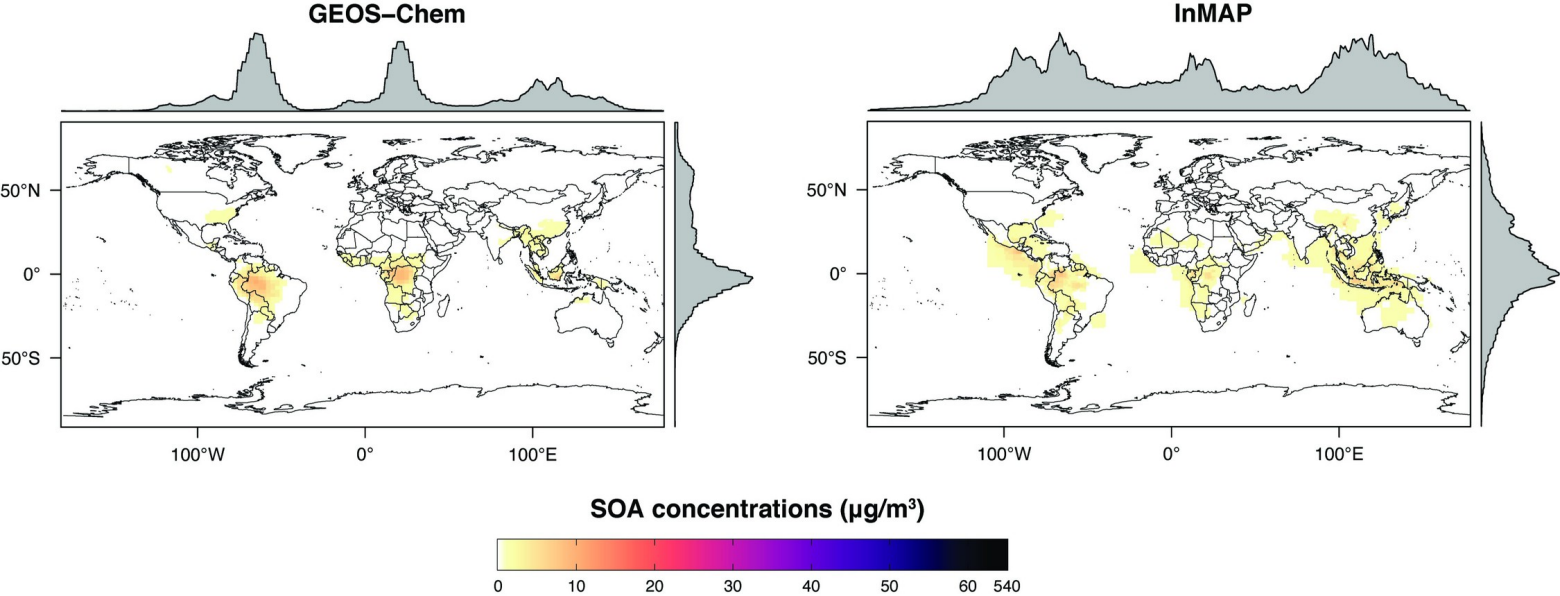
Global InMAP and GEOS-Chem annual-average ground-level SOA concentrations.

**Fig 10:**
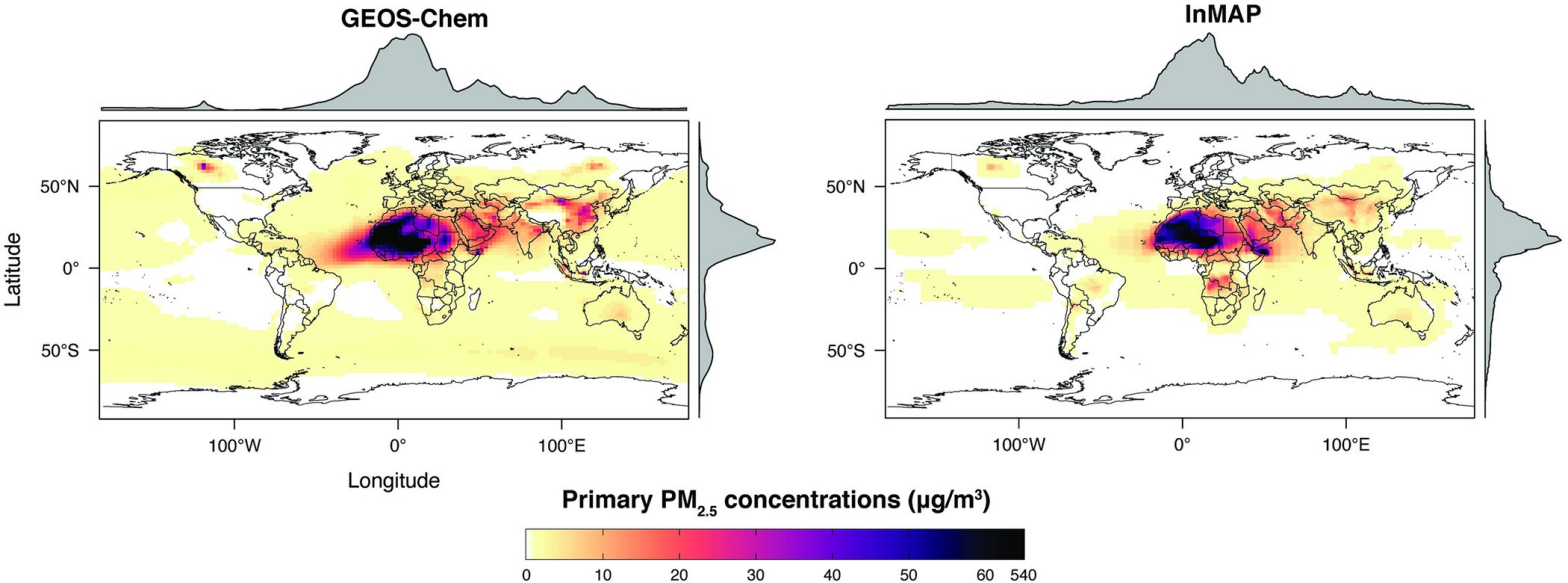
Global InMAP and GEOS-Chem annual-average ground-level primary PM_2.5_ concentrations.

We also compared annual-average predicted concentrations from the Global InMAP simulation to annual-average measurements of pSO_4_, pNO_3_, and pNH_4_ globally (Figs 3–8). The Global InMAP simulation predicted these components well (NME: 48%–66%; R^2^: 0.25–0.46) and was generally biased low against measurements for pNO_3_ (especially in areas with pNO_3_ >2 μg m^-3^), and high for pSO_4_. Because the Global InMAP simulation did not have a strong low bias against secondary inorganic PM_2.5_ measurements, it is likely that much of the low bias of the Global InMAP simulation against total PM_2.5_ measurements arose from its prediction of primary PM_2.5_ concentrations (see Fig 9), which have a 4.05 μg m^-3^ lower population-weighted mean concentration globally compared to GEOS-Chem (see Table D in S1 Appendix). However, measurement data for SOA and primary PM_2.5_ concentrations were not available at the evaluation sites (see Figs 9 and 10 for ground-level concentrations of these species, and Table D in S1 Appendix for population-weighted concentrations). Against pollutant concentration estimates that make use of satellite data from Li *et al*. [50], both the GEOS-Chem and Global InMAP simulations underpredict population-weighted concentrations of all PM_2.5_ species, especially SOA concentrations (see Table D in S1 Appendix), consistent with prior findings for other chemical transport models [50].

We also compared the GEOS-Chem simulation against the same measurement data, to contextualize the Global InMAP results. The GEOS-Chem simulation predicted total PM_2.5_ measurements with an R^2^ of 0.55. For comparison, a GEOS-Chem simulation that used the same code and emissions [29], but estimated PM_2.5_ concentrations derived from simulated aerosol optical depth, reported an R^2^ of 0.61, when using a more comprehensive measurement dataset and averaging results across years 2010–2018 instead of just 2016.

Both the Global InMAP and the GEOS-Chem simulations predicted lower annual-average total PM_2.5_ concentrations than were observed. For all species and regions, the direction of bias against measurements was the same for the Global InMAP simulation as for the GEOS-Chem simulation. This suggests that some of the bias observed in the Global InMAP simulation was inherited from the bias in GEOS-Chem and/or in the simulation inputs such as the emissions inventories. If that was the case, then future improvements to the GEOS-Chem model and to the emission inventories used here could further reduce Global InMAP biases.

The Global InMAP simulation broadly reproduced spatial patterns of pollutant concentrations predicted by GEOS-Chem (see Table D in S1 Appendix for population-weighted concentrations, and Table F in S1 Appendix for region descriptions). However, there were some features present in the GEOS-Chem simulation that were not captured by the Global InMAP simulation. Such features included high annual-average PM_2.5_ concentrations from biomass burning, including the Alberta fires, crop burning in the Indo-Gangetic Plain, peatland fires in Singapore and Malaysia, and burning in Siberia. InMAP may have underpredicted PM_2.5_ concentrations from biomass burning relative to the GEOS-Chem simulation because it uses annual-average representations of pollutant emissions, fate, and transport. Across Western China, the Global InMAP simulation tended to misrepresent the spatial patterns provided by the parent GEOS-Chem simulation for both primary and secondary PM_2.5_, including high concentrations over the Himalayas and Sichuan Basin, and low concentrations in surrounding areas. This may suggest that the annual-average advection scheme used by InMAP does not yet adequately capture complex air flows over steep terrain.

### Evaluation of predicted responses to changes in emissions

The major intended use of InMAP is to estimate the changes in PM_2.5_ concentrations for given scenarios of emission changes. Therefore, its ability to reproduce the changes predicted by the original CTM could be considered its most important attribute, more important than its ability to reproduce current absolute concentrations. However, InMAP is designed to predict PM_2.5_ concentrations with high spatial resolution in urban areas, whereas GEOS-Chem is designed to predict global chemical transport and runs at comparatively low resolution. Directly comparing the two models requires re-gridding the higher-resolution Global InMAP results to match the lower-resolution GEOS-Chem results, which cancels out predictive advantages Global InMAP might gain from its use of higher spatial resolution. Therefore, results in this section could be considered a conservative evaluation of Global InMAP’s predictive performance.

**Fig 11:**
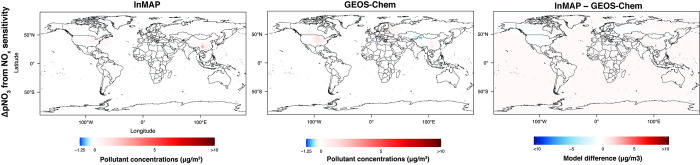
Comparison between Global InMAP and GEOS-Chem for predicting changes in pNO_3_ concentrations from a 100% increase in NO_x_ emissions from road transportation.

**Fig 12:**
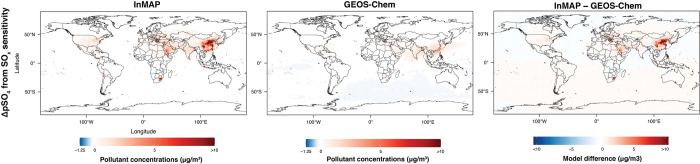
Comparison between Global InMAP and GEOS-Chem for predicting changes in pSO_4_ concentrations from a 100% increase in SO_x_ emissions from power generation.

**Fig 13:**
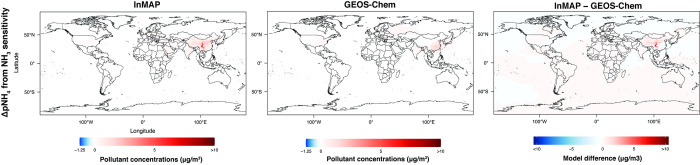
Comparison between Global InMAP and GEOS-Chem for predicting changes in pNH_4_ concentrations from a 100% increase in NH_3_ emissions from agricultural soils.

Figs 11–13 show annual-average pollutant concentration increments predicted by the GEOS-Chem and Global InMAP simulations for increases in SO_x_ emissions from power generation, NH_3_ emissions from agricultural soils, and NO_x_ emissions from road transportation. When regridding Global InMAP predictions to the GEOS-Chem grid, we found that Global InMAP reproduced the GEOS-Chem results with an average area-weighted NME of 118–182% and an average area-weighted NMB of 59–121% (see Table 2; Table E in S1 Appendix). For the NO_x_ and NH_3_ emissions scenarios, Global InMAP exhibited better performance against GEOS-Chem on a population-weighted basis than on an area-weighted basis, including over different regions. For the SO_x_ emissions scenario, Global InMAP exhibited the lowest overall performance against the GEOS-Chem simulation, having overpredicted changes in pSO_4_ concentrations in populated regions such as East Asia and Africa (see Table E in S1 Appendix). Although Global InMAP did not perform well against measurements in South Asia (see Table C in S1 Appendix), for changes in pollutant concentrations, Global InMAP reproduces GEOS-Chem concentrations across South Asia with population-weighted NME of 44–59% and NMB of 5–24%, supporting the utility of the model for policy assessment in the region.

**Table 2:** Area- and population-weighted (wtd.) normalized mean bias (NMB) and error (NME) for Global InMAP predicted changes in concentrations against changes in concentrations from GEOS-Chem or US InMAP, arising from scenarios of changes in emissions. Positive bias indicates that Global InMAP has higher average concentration changes than the other model.

Model comparison	Scenario	Weighting	NME (%)	NMB (%)
Global InMAP against GEOS-Chem	NH_3_ increase from agricultural soils	area-wtd.	118.2	58.7
		population-wtd.	81.8	58.7
	NO_x_ increase from road transportation	area-wtd.	180.7	96.2
		population-wtd.	106.7	48.9
	SO_x_ increase from power generation	area-wtd.	181.3	120.7
		population-wtd.	275.4	216.9
Global InMAP against US InMAP	Coal-powered electricity	area-wtd.	38.4	-18.8
		population-wtd.	38.7	-10.5
	Gasoline passenger vehicles	area-wtd.	48.4	-23.0
		population-wtd.	48.8	-46.7

**Fig 14:**
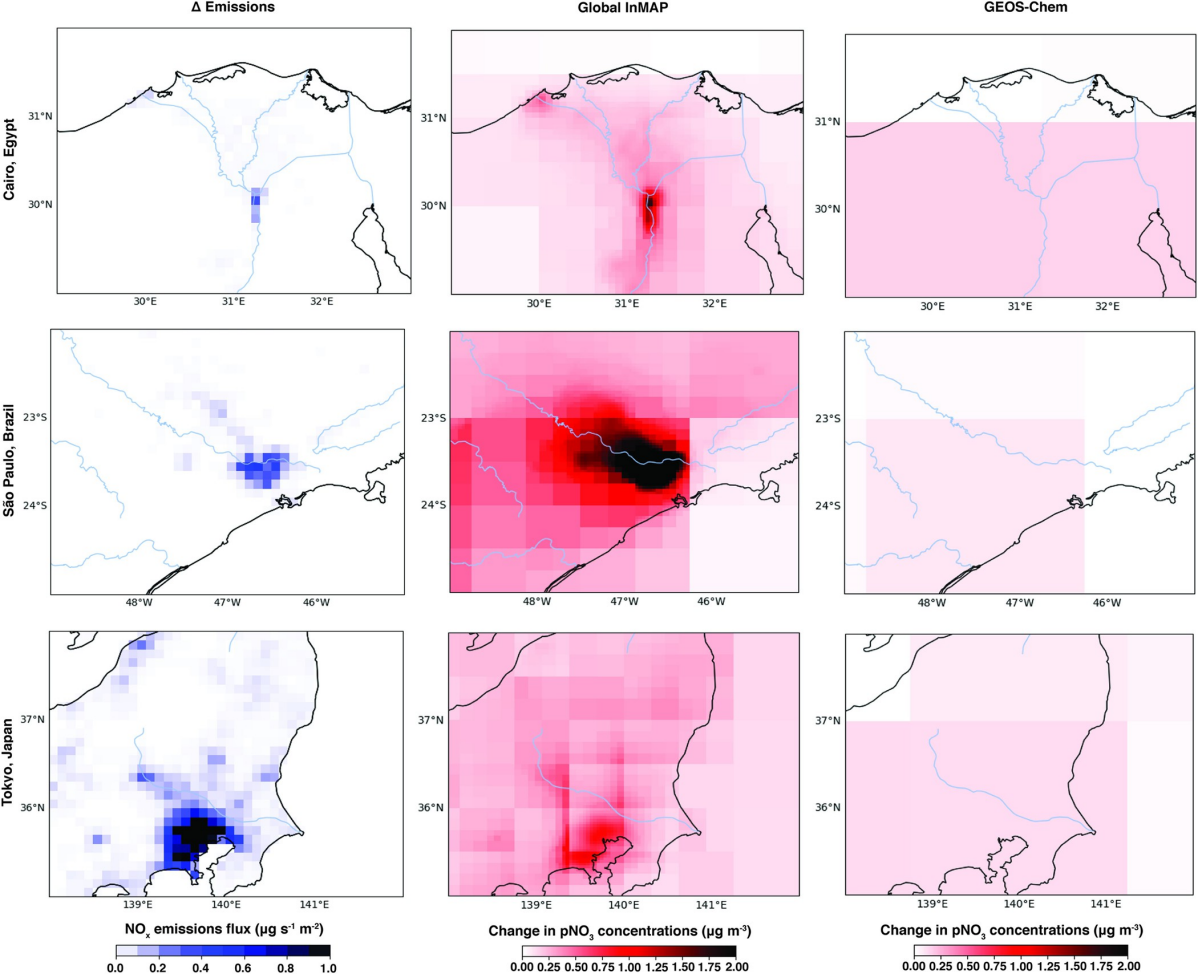
*First column*: 100% increase in NO_x_ emissions from road transport across Cairo, São Paulo, and Tokyo. *Second and third column*: Resulting changes in pNO_3_ concentrations predicted by the Global InMAP and the GEOS-Chem simulations. For each map, blue lines indicate rivers and black lines indicate land borders.

The Global InMAP simulations predicted greater variability in concentration changes over urban areas than the 2° × 2.5° GEOS-Chem simulations for the same emissions scenarios, owing to its higher resolution grid. Fig 14 compares the pNO_3_ concentration changes over Cairo, São Paulo, and Tokyo (the largest cities in Africa, South America, and Asia [64]) for the NO_x_ perturbation scenario as predicted by Global InMAP and GEOS-Chem. The urban-scale fidelity of Global InMAP in cities worldwide can capture the changes in concentrations one would expect from local pollution sources, whereas the coarser global model cannot. Higher resolution GEOS-Chem simulations that resolve intra-urban gradients would be even more computationally expensive than the GEOS-Chem simulations performed here [57].

Global InMAP predicted similar spatial patterns and magnitudes of changes in pollutant concentrations as did US InMAP for a given emissions perturbation (see S10 Fig), with NME and NMB within ± 50% for both scenarios considered (see Table 2). This demonstrated consistency between the InMAP versions derived from WRF-Chem and GEOS-Chem inputs, suggesting that no major errors were introduced in the Global InMAP model development (see Table A in S1 Appendix; Tessum *et al*. [5]). For InMAP applications focusing only on the United States, continued use of US InMAP is warranted, as the WRF-Chem simulation used to parameterize US InMAP provides higher spatial resolution than does the nested GEOS-Chem simulation employed for Global InMAP.

## Discussion

Here, we extended InMAP, a reduced-complexity air quality model originally developed for use in the United States, to simulate a global-through-urban spatial domain. InMAP is designed to supplement rather than supplant state-of-the-science tools such as GEOS-Chem or other global models, *e*.*g*., for cases in which (i) resources to implement a CTM are unavailable, (ii) numerous simulations are needed to evaluate a large variety of policy scenarios, or (iii) the primary need is initial assessment and screening. The accuracy of InMAP is not as high as with a CTM (e.g., here, a normalized mean error of 62% (InMAP) versus 41% (GEOS-Chem)), yet for many scientific and policy questions lacking readily-available CTM-quality results, InMAP provides useful information.

Global InMAP requires relatively low computational resources, allowing annual-average simulations to readily be run on a desktop computer rather than a supercomputer, and take a few hours rather than days. For example, compared to the global GEOS-Chem simulation described here, the Global InMAP simulation was 12× faster at predicting total annual-average PM_2.5_ concentrations, despite the Global InMAP simulation having 66× higher population-weighted average spatial resolution (as low as ~4km).

As expected, the expedience of Global InMAP comes at the expense of lower predictive accuracy compared to a comprehensive CTM. This Global InMAP simulation is biased low against measurements for total PM_2.5_ across all regions. Among species, it is biased high against measurements of pSO_4_ and pNH_4_, and low against measurements of pNO_3_. The low computational resource requirements make Global InMAP particularly well-suited to applications where hundreds of policy scenarios are evaluated, as is often done using reduced-complexity models for the United States [8,11,65], or when no other air quality models are available at the urban scale. In places with higher population and pollution exposure than the United States, there is even more potential for a reduced-complexity model such as Global InMAP to inform impactful policy decisions. Global InMAP may be important for informing preliminary hypotheses about policy decisions in its early stages (*e*.*g*., “What is the best location to site a new facility that may be a major pollution source?”), allowing computational resources to be used instead for CTMs at a later stage to check consistency with the findings.

Global InMAP performance varies regionally, both against measurements and against GEOS-Chem when estimating changes in concentrations. Across South Asia, Global InMAP performs poorly against measurements, but for the changes in emissions considered here, Global InMAP predicts fairly similar changes in concentrations to GEOS-Chem. Against measurements, Global InMAP is generally most accurate in Oceania, North America, and Europe, and tends to perform worse in places where GEOS-Chem also performs poorly (*e*.*g*., South America, where both GEOS-Chem and Global InMAP exhibit a weak negative linear correlation against measurements). This suggests that Global InMAP performance in those regions may improve based on future advancements in emission inventories or GEOS-Chem model inputs. This Global InMAP version was developed using outputs from GEOS-Chem v11-01f, whereas GEOS-Chem v13.3.3 is currently released. GEOS-Chem v13.3.3 has many improvements over v11-01f, including the ability to easily define high resolution global nested grids. As well as potentially reducing bias, higher resolution GEOS-Chem inputs to Global InMAP will reduce artefacts from coarse inputs, such as those observed over cities in Fig 14. Furthermore, higher resolution global meteorology inputs are becoming increasingly available, and may further be used to improve Global InMAP performance.

By directly estimating annual-average PM_2.5_ concentrations at high spatial resolution, Global InMAP is configured to easily estimate changes in human exposure and health impacts. When estimating human health effects of emissions changes, there will also be sizeable uncertainties from estimating the emissions changes themselves and from the concentration-response function employed [66]. Global InMAP errors should thus be contextualized with those uncertainties in mind. For example, cost-benefit analyses typically make use of highly uncertain mortality estimation and economic valuation to arrive at air quality-related costs and benefits [67]. For the United States, a previous study [6] found that the largest source of uncertainty in estimating monetized PM_2.5_ health impacts was the economic valuation of premature mortality, followed by the concentration-response function, whereas uncertainty in PM_2.5_ concentrations from the choice of air quality model was the smallest source of uncertainty considered. Since uncertainty in the air pollution model is not the largest source of uncertainty in many contexts, there are many cases in which a reduced-complexity model (RCM) can deliver useful information sufficient for policy analyses.

That conclusion especially applies to the many cases where resources may exist to run an RCM but not to establish and run a conventional CTM. Indeed, there are many cases in which a CTM simulation is infeasible, yet an RCM or other approach could provide some information. As mentioned above, InMAP is not a replacement for a CTM; instead, it provides screening-level information, results for questions that would involve too many model runs to use a CTM, or results that would be otherwise infeasible. By providing a global, open source, air quality model with high spatial resolution and low computational requirements, we hope to facilitate the wide practice of air pollution policy assessment worldwide.

## Supporting information

S1 Appendix (DOCX)

S1 Fig InMAP grid details. Detail of the Global InMAP horizontal computational grid over West Africa, Central America, and Europe for illustration. Grid cells are as small as 0.04° × 0.03° (~4 km length) in areas with a higher population such as Lagos in Nigeria, San Salvador in El Salvador, and London in the United Kingdom. Grid cells are as large as 5° × 4° (~500 km length) in places with a lower population, such as across the Atlantic Ocean.
(TIFF)

S2 Fig Total concentrations against measurements, including outliers. InMAP and GEOS-Chem annual-average primary PM_2.5_ concentrations against measurements, including outliers (above 100 μg m^-3^). Pop-wtd: Population-weighted metrics.
(TIFF)

S3 Fig Model performance for Africa. Performance of Global InMAP and GEOS-Chem simulations against total annual-average PM_2.5_ measurements for Africa. Dots on each map show measurement site locations, whose color corresponds to the model-measurement difference in PM_2.5_ concentrations.
(TIFF)

S4 Fig Model performance for East Asia. Performance of Global InMAP and GEOS-Chem simulations against total annual-average PM_2.5_ measurements for East Asia. Dots on each map show measurement site locations, whose color corresponds to the model-measurement difference in PM_2.5_ concentrations.
(TIFF)

S5 Fig Model performance for South Asia. Performance of Global InMAP and GEOS-Chem simulations against total annual-average PM_2.5_ measurements for South Asia. Dots on each map show measurement site locations, whose color corresponds to the model-measurement difference in PM_2.5_ concentrations.
(TIFF)

S6 Fig Model performance for Europe. Performance of Global InMAP and GEOS-Chem simulations against total annual-average PM_2.5_ measurements for Europe. Dots on each map show measurement site locations, whose color corresponds to the model-measurement difference in PM_2.5_ concentrations.
(TIFF)

S7 Fig Model performance for North and Central America. Performance of Global InMAP and GEOS-Chem simulations against total annual-average PM_2.5_ measurements for North and Central America. Dots on each map show measurement site locations, whose color corresponds to the model-measurement difference in PM_2.5_ concentrations.
(TIFF)

S8 Fig Model performance for Oceania. Performance of Global InMAP and GEOS-Chem simulations against total annual-average PM_2.5_ measurements for Oceania. Dots on each map show measurement site locations, whose color corresponds to the model-measurement difference in PM_2.5_ concentrations.
(TIFF)

S9 Fig Model performance for South America. Performance of Global InMAP and GEOS-Chem simulations against total annual-average PM_2.5_ measurements for South America. Dots on each map show measurement site locations, whose color corresponds to the model-measurement difference in PM_2.5_ concentrations.
(TIFF)

S10 Fig Comparison between Global and US InMAP. Changes in Total PM_2.5_ concentrations from road vehicle emissions and from power generation emissions as predicted by Global InMAP (which has GEOS-Chem preprocessor inputs) alongside US InMAP (which has WRF-Chem preprocessor inputs).
(TIFF)

## References

[1] Landrigan PJ, Fuller R, Acosta NJ, Adeyi O, Arnold R, Baldé AB, et al. The Lancet Commission on pollution and health. The Lancet. 2018 Feb 3;391(10119):462–512. 10.1016/S0140-6736(17)32345-0 29056410

[2] Burnett R, Chen H, Szyszkowicz M, Fann N, Hubbell B, Pope CA, et al. Global estimates of mortality associated with long-term exposure to outdoor fine particulate matter. Proceedings of the National Academy of Sciences. 2018 Sep 18;115(38):9592–7. doi: 10.1073/pnas.1803222115 30181279 PMC6156628

[3] Silva RA, Adelman Z, Fry MM, West JJ. The impact of individual anthropogenic emissions sectors on the global burden of human mortality due to ambient air pollution. Environmental health perspectives. 2016 Nov;124(11):1776–84. doi: 10.1289/EHP177 27177206 PMC5089880

[4] Lelieveld J, Evans JS, Fnais M, Giannadaki D, Pozzer A. The contribution of outdoor air pollution sources to premature mortality on a global scale. Nature. 2015 Sep;525(7569):367–71. doi: 10.1038/nature15371 26381985

[5] Tessum CW, Hill JD, Marshall JD. InMAP: A model for air pollution interventions. PloS one. 2017 Apr 19;12(4):e0176131. doi: 10.1371/journal.pone.0176131 28423049 PMC5397056

[6] Goodkind AL, Tessum CW, Coggins JS, Hill JD, Marshall JD. Fine-scale damage estimates of particulate matter air pollution reveal opportunities for location-specific mitigation of emissions. Proceedings of the National Academy of Sciences. 2019 Apr 30;116(18):8775–80. doi: 10.1073/pnas.1816102116 30962364 PMC6500143

[7] Tessum CW, Apte JS, Goodkind AL, Muller NZ, Mullins KA, Paolella DA, et al. Inequity in consumption of goods and services adds to racial–ethnic disparities in air pollution exposure. Proceedings of the National Academy of Sciences. 2019 Mar 26;116(13):6001–6. doi: 10.1073/pnas.1818859116 30858319 PMC6442600

[8] Thakrar SK, Balasubramanian S, Adams PJ, Azevedo IM, Muller NZ, Pandis SN, et al. Reducing mortality from air pollution in the United States by targeting specific emission sources. Environmental Science & Technology Letters. 2020 Jul 15;7(9):639–45.

[9] Liu L, Hwang T, Lee S, Ouyang Y, Lee B, Smith SJ, et al. Health and climate impacts of future United States land freight modelled with global-to-urban models. Nature Sustainability. 2019 Feb;2(2):105–12.

[10] Thind MPS, Tessum CW, Azevedo IL, Marshall JD. Fine particulate air pollution from electricity generation in the US: Health impacts by race, income, and geography. Environmental Science & Technology. 2019; 53.23:14010–14019. 31746196 10.1021/acs.est.9b02527

[11] Hill J, Goodkind A, Tessum C, Thakrar S, Tilman D, Polasky S, et al. Air-quality-related health damages of maize. Nature Sustainability. 2019 May;2(5):397–403.

[12] Heo J, Adams PJ, Gao HO. Reduced-form modeling of public health impacts of inorganic PM2. 5 and precursor emissions. Atmospheric Environment. 2016; Jul 1;137:80–9.

[13] Muller NZ. Boosting GDP growth by accounting for the environment. Science. 2014 Aug 22;345(6199):873–4. doi: 10.1126/science.1253506 25146270

[14] US Environmental Protection Agency. User’s manual for the co-benefits risk assessment (COBRA), 2018.

[15] Lee CJ, Martin RV, Henze DK, Brauer M, Cohen A, Donkelaar AV. Response of global particulate-matter-related mortality to changes in local precursor emissions. Environmental Science & Technology. 2015 Apr 7;49(7):4335–44. doi: 10.1021/acs.est.5b00873 25730303

[16] GBD Results Tool. Accessed: 25^th^ January 2021. http://ghdx.healthdata.org/gbd-results-tool.

[17] Maps of GEOS-Chem User Groups. Accessed: 25^th^ January 2021. http://acmg.seas.harvard.edu/geos/geos_people.html.

[18] Van Dingenen R, Dentener F, Crippa M, Leitao J, Marmer E, Rao S, et al. TM5-FASST: a global atmospheric source–receptor model for rapid impact analysis of emission changes on air quality and short-lived climate pollutants. Atmospheric Chemistry and Physics. 2018 Nov 13;18(21):16173–211.

[19] Henze DK, Hakami A, Seinfeld JH. Development of the adjoint of GEOS-Chem. Atmospheric Chemistry and Physics. 2007 May 11;7(9):2413–33.

[20] Amann M, Bertok I, Borken-Kleefeld J, Cofala J, Heyes C, Höglund-Isaksson L, et al. Cost-effective control of air quality and greenhouse gases in Europe: Modeling and policy applications. Environmental Modelling & Software. 2011 Dec 1;26(12):1489–501.

[21] Oberschelp C, Pfister S, Hellweg S. Globally regionalized monthly life cycle impact assessment of particulate matter. Environmental Science & Technology. 2020 Nov 23;54(24):16028–38. doi: 10.1021/acs.est.0c05691 33226786

[22] Gilmore EA, Heo J, Muller NZ, Tessum CW, Hill JD, Marshall JD, et al. An inter-comparison of the social costs of air quality from reduced-complexity models. Environmental Research Letters. 2019 Jul 9;14(7):074016.

[23] Bey I, Jacob DJ, Yantosca RM, Logan JA, Field BD, Fiore AM, et al. Global modeling of tropospheric chemistry with assimilated meteorology: Model description and evaluation. Journal of Geophysical Research: Atmospheres. 2001 Oct 16;106(D19):23073–95.

[24] Grell GA, Peckham SE, Schmitz R, McKeen SA, Frost G, Skamarock WC, et al. Fully coupled “online” chemistry within the WRF model. Atmospheric Environment. 2005 Dec 1;39(37):6957–75.

[25] Tessum CW, Hill JD, Marshall JD. Twelve-month, 12 km resolution North American WRF-Chem v3. 4 air quality simulation: performance evaluation. Geoscientific Model Development. 2015 Apr 7;8(4):957–73.

[26] Gridded Population of the World, Version 4 (GPWv4): National Identifier Grid. Palisades, NY: NASA Socioeconomic Data and Applications Center (SEDAC). 10.7927/H41V5BX1.

[27] Park RJ, Jacob DJ, Field BD, Yantosca RM, Chin M. Natural and transboundary pollution influences on sulfate‐nitrate‐ammonium aerosols in the United States: Implications for policy. Journal of Geophysical Research: Atmospheres. 2004 Aug 16;109(D15).

[28] Gelaro R, McCarty W, Suárez MJ, Todling R, Molod A, Takacs L, et al. The modern-era retrospective analysis for research and applications, version 2 (MERRA-2). Journal of climate. 2017 Jul 15;30(14):5419–54. doi: 10.1175/JCLI-D-16-0758.1 32020988 PMC6999672

[29] Hammer MS, van Donkelaar A, Li C, Lyapustin A, Sayer AM, Hsu NC, et al. Global estimates and long-term trends of fine particulate matter concentrations (1998–2018). Environmental Science & Technology. 2020 Jun 3;54(13):7879–90. doi: 10.1021/acs.est.0c01764 32491847

[30] Pye HOT; Seinfeld JH. A global perspective on aerosol from low-volatility organic compounds. Atmospheric Chemistry and Physics 2010;10(9):4377–4401.

[31] Pye HO, Chan AW, Barkley MP, Seinfeld JH. Global modeling of organic aerosol: the importance of reactive nitrogen (NOx and NO3). Atmospheric Chemistry and Physics. 2010 Nov 30;10(22):11261–76.

[32] Wang YX, McElroy MB, Jacob DJ, Yantosca RM. A nested grid formulation for chemical transport over Asia: Applications to CO. Journal of Geophysical Research: Atmospheres. 2004 Nov 27;109(D22).

[33] Keller CA, Long MS, Yantosca RM, Da Silva AM, Pawson S, Jacob DJ. HEMCO v1. 0: a versatile, ESMF-compliant component for calculating emissions in atmospheric models. Geoscientific Model Development. 2014 Jul 14;7(4):1409–17.

[34] Crippa M, Guizzardi D, Muntean M, Schaaf E, Dentener F, Van Aardenne JA, et al. Gridded emissions of air pollutants for the period 1970–2012 within EDGAR v4. 3.2. Earth Syst. Sci. Data. 2018 Oct 26;10(4):1987–2013.

[35] Kuhns H, Green M, Etyemezian V, Watson J, Pitchford M. Big bend regional aerosol and visibility observational (BRAVO) study emissions inventory. Report prepared for BRAVO Steering Committee, Desert Research Institute, Las Vegas, Nevada. 2003.

[36] Vestreng V. Emission data reported to UNECE/EMEP: Quality assurance and trend analysis & presentation of WebDab: MSC-W status report 2002.

[37] Li M, Zhang Q, Kurokawa JI, Woo JH, He K, Lu Z, et al. MIX: a mosaic Asian anthropogenic emission inventory under the international collaboration framework of the MICS-Asia and HTAP. Atmospheric Chemistry and Physics. 2017 Jan 20;17(2):935–63.

[38] Zheng B, Huo H, Zhang Q, Yao ZL, Wang XT, Yang XF, et al. High-resolution mapping of vehicle emissions in China in 2008. Atmospheric Chemistry and Physics. 2014 Sep 17;14(18):9787–805.

[39] Lu Z, Zhang Q, Streets DG. Sulfur dioxide and primary carbonaceous aerosol emissions in China and India, 1996–2010. Atmospheric Chemistry and Physics. 2011 Sep 23;11(18):9839–64.

[40] Stettler ME, Eastham S, Barrett SR. Air quality and public health impacts of UK airports. Part I: Emissions. Atmospheric environment. 2011 Oct 1;45(31):5415–24.

[41] Holmes CD, Prather MJ, Vinken GC. The climate impact of ship NOx emissions: an improved estimate accounting for plume chemistry. Atmospheric Chemistry and Physics. 2014 Jul 4;14(13):6801–12.

[42] Schultz MG, Heil A, Hoelzemann JJ, Spessa A, Thonicke K, Goldammer JG, et al. Global wildland fire emissions from 1960 to 2000. Global Biogeochemical Cycles. 2008 Jun;22(2).

[43] Giglio L, Randerson JT, Van Der Werf GR. Analysis of daily, monthly, and annual burned area using the fourth‐generation global fire emissions database (GFED4). Journal of Geophysical Research: Biogeosciences. 2013 Mar;118(1):317–28.

[44] Ge C, Wang J, Carn S, Yang K, Ginoux P, Krotkov N. Satellite‐based global volcanic SO2 emissions and sulfate direct radiative forcing during 2005–2012. Journal of Geophysical Research: Atmospheres. 2016 Apr 16;121(7):3446–64.

[45] Hudman RC, Moore NE, Mebust AK, Martin RV, Russell AR, Valin LC, et al. Steps towards a mechanistic model of global soil nitric oxide emissions: implementation and space based-constraints. Atmospheric Chemistry & Physics. 2012; 12(16):7779–7795.

[46] Guenther AB, Jiang Xiaoyan, Heald CL, Sakulyanontvittaya T, Duhl Ti any, Emmons LK, et al. The Model of Emissions of Gases and Aerosols from Nature version 2.1 (MEGAN2. 1): an extended and updated framework for modeling biogenic emissions. Geoscientific Model Development. 2012; 5(6):1471–1492.

[47] Zender CS, Bian H, Newman D. Mineral Dust Entrainment and Deposition (DEAD) model: Description and 1990s dust climatology. Journal of Geophysical Research: Atmospheres. 2003 Jul 27;108(D14).

[48] Ahmadov R, McKeen SA, Robinson AL, Bahreini R, Middlebrook AM, De Gouw JA, et al. A volatility basis set model for summertime secondary organic aerosols over the eastern United States in 2006. Journal of Geophysical Research: Atmospheres. 2012 Mar 27;117(D6).

[49] Philip S, Martin RV, Pierce JR, Jimenez JL, Zhang Q, Canagaratna MR, et al. Spatially and seasonally resolved estimate of the ratio of organic mass to organic carbon. Atmospheric Environment. 2014 Apr 1;87:34–40.

[50] Li C, Martin RV, van Donkelaar A, Boys BL, Hammer MS, Xu JW, et al. Trends in chemical composition of global and regional population-weighted fine particulate matter estimated for 25 years. Environmental science & technology. 2017 Oct 3;51(19):11185–95. doi: 10.1021/acs.est.7b02530 28891283

[51] Homer C, Huang C, Yang L, Wylie B, Coan M. Development of a 2001 national land-cover database for the United States. Photogrammetric Engineering & Remote Sensing. 2004 Jul 1;70(7):829–40.

[52] GEOS-Chem Wiki. Olson land map. Accessed: 25^th^ January 2021. http://wiki.seas.harvard.edu/geos-chem/index.php/Olson_land_map.

[53] Dennis R, Fox T, Fuentes M, Gilliland A, Hanna S, Hogrefe C, et al. A framework for evaluating regional-scale numerical photochemical modeling systems. Environmental Fluid Mechanics. 2010 Aug;10(4):471–89. doi: 10.1007/s10652-009-9163-2 21461126 PMC3066450

[54] Diao M, Holloway T, Choi S, O’Neill SM, Al-Hamdan MZ, Van Donkelaar A, et al. Methods, availability, and applications of PM2. 5 exposure estimates derived from ground measurements, satellite, and atmospheric models. Journal of the Air & Waste Management Association. 2019 Dec 2;69(12):1391–414. 31526242 10.1080/10962247.2019.1668498 PMC7072999

[55] Emery C, Liu Z, Russell AG, Odman MT, Yarwood G, Kumar N. Recommendations on statistics and benchmarks to assess photochemical model performance. Journal of the Air & Waste Management Association. 2017 Apr 27;67(5):582–98. doi: 10.1080/10962247.2016.1265027 27960634

[56] Boylan JW, Russell AG. PM and light extinction model performance metrics, goals, and criteria for three-dimensional air quality models. Atmospheric environment. 2006 Aug 1;40(26):4946–59.

[57] Eastham SD, Long MS, Keller CA, Lundgren E, Yantosca RM, Zhuang J, et al. GEOS-Chem High Performance (GCHP v11-02c): a next-generation implementation of the GEOS-Chem chemical transport model for massively parallel applications. Geoscientific Model Development. 2018; 11(7):2941–2953.

[58] Huneeus N, van der Gon HD, Castesana P, Menares C, Granier C, Granier L, et al. Evaluation of anthropogenic air pollutant emission inventories for South America at national and city scale. Atmospheric Environment. 2020; 235:117606.

[59] Van Damme M, Clarisse L, Whitburn S, Hadji-Lazaro J, Hurtmans D, Clerbaux C, et al. Industrial and agricultural ammonia point sources exposed. Nature. 2018 Dec;564(7734):99–103. doi: 10.1038/s41586-018-0747-1 30518888

[60] Akherati A, He Y, Coggon MM, Koss AR, Hodshire AL, Sekimoto K, et al. Oxygenated aromatic compounds are important precursors of secondary organic aerosol in biomass-burning emissions. Environmental Science & Technology. 2020 Jun 19;54(14):8568–79. doi: 10.1021/acs.est.0c01345 32559089

[61] Shaik DS, Kant Y, Mitra D, Singh A, Chandola HC, Sateesh M, et al. Impact of biomass burning on regional aerosol optical properties: A case study over northern India. Journal of environmental management. 2019;244:328–343. doi: 10.1016/j.jenvman.2019.04.025 31129465

[62] Rastogi N, Singh A, Sarin MM, Singh D. Temporal variability of primary and secondary aerosols over northern India: Impact of biomass burning emissions. Atmospheric environment. 2016;125:396–403.

[63] Zheng B, Zhang Q, Zhang Y, He KB, Wang K, Zheng GJ, et al. Heterogeneous chemistry: a mechanism missing in current models to explain secondary inorganic aerosol formation during the January 2013 haze episode in North China. Atmospheric Chemistry and Physics. 2015 Feb 25;15(4):2031–49.

[64] United Nations, Department of Economic and Social Affairs, Population Division. World Urbanization Prospects: The 2018 Revision (ST/ESA/SER.A/420). 2014. New York: United Nations.

[65] Muller NZ, Mendelsohn R. Efficient pollution regulation: getting the prices right. American Economic Review. 2009 Dec;99(5):1714–39.

[66] Coffman E, Burnett RT, Sacks JD. Quantitative Characterization of Uncertainty in the Concentration–Response Relationship between Long-Term PM2. 5 Exposure and Mortality at Low Concentrations. Environmental Science & Technology. 2020 Jul 23;54(16):10191–200. doi: 10.1021/acs.est.0c02770 32702976 PMC8167809

[67] US Environmental Protection Agency. Regulatory impact analysis for the final revisions to the national ambient air quality standards for particulate matter. 2012 Dec;EPA-452/R-12-005.

