# Supplementary material for: Global, high-resolution, reduced-complexity air quality modeling for PM2.5 using InMAP (Intervention Model for Air Pollution)
Source: PLoS One. 2022 May 25;17(5):e0268714. doi: 10.1371/journal.pone.0268714 (PMC9132322; doi:10.1371/journal.pone.0268714)
Supplement: S1 Appendix — (DOCX) [file pone.0268714.s001.docx]

Global, high-resolution, reduced-complexity air quality modeling for PM_2.5_ using InMAP (Intervention Model for Air Pollution)

Short title: Global, reduced-complexity air quality modeling

Sumil K. Thakrar^1,2*^, Christopher W. Tessum^3^, Joshua S. Apte^4,5^, Srinidhi Balasubramanian^1^, Dylan B. Millet^6^, Spyros N. Pandis^7,8^, Julian D. Marshall^9^, Jason D. Hill^1*^.

^1^Department of Bioproducts & Biosystems Engineering, University of Minnesota, St Paul, Minnesota, United States of America.

^2^Department of Applied Economics, University of Minnesota, St Paul, Minnesota, United States of America.

^3^Department of Civil and Environmental Engineering, University of Illinois at Urbana−Champaign, Urbana, Illinois, United States of America.

^4^Department of Civil and Environmental Engineering, University of California, Berkeley, Berkeley, California, United States of America.

^5^School of Public Health, University of California, Berkeley, Berkeley, California, United States of America.

^6^Department of Soil, Water, and Climate, University of Minnesota, St Paul, Minnesota, United States of America.

^7^Department of Chemical Engineering, Carnegie Mellon University, Pittsburgh, Pennsylvania, United States of America.

^8^Department of Chemical Engineering, University of Patras, Patras, Greece.

^9^Department of Civil and Environmental Engineering, University of Washington, Seattle, Washington, United States of America.

* Corresponding author

Email: [sthakrar@umn.edu](mailto:sthakrar@umn.edu) (SKT)

Supporting Information

**Text A. Measurement data description.**

Ground-level measurements of total PM_2.5_, pNH_4_, pNO_3_, and pSO_4_ concentrations across year 2016 were compiled from the World Health Organization database and supplemented with additional measurements from other official channels such as governmental and non-governmental agencies (see Table S2). Included measurements were vetted according to quality control criteria, including those used by the 2012 United States National Ambient Air Quality Standards. Only measurements that directly measured PM_2.5_ were included; PM_10_ measurements that were converted to PM_2.5_ were excluded. Further, data without correct latitude and longitude were excluded. Global InMAP directly estimates annual-average pollutant concentrations, so measurement data from each monitoring site were averaged across the year. To avoid temporal biases across the day, all measurement data were averaged daily values of pollutant concentrations. To avoid seasonal biases, measurements had to be reported for at least 75% of days in the year from each monitoring site included in our dataset.

After vetting, the final dataset of annual-average pollutant concentrations included ~1,700 total PM_2.5_ data points across 62 countries; 171 pNH_4_ data points across 1 country (the US); 334 pNO_3_ data points across 4 countries, and 385 pSO_4_ data points across 12 countries. The final dataset is provided in an online repository (doi:10.5281/zenodo.4641948).

**Text B. Performance metric and criteria descriptions.**

Normalized mean bias and error (NMB and NME), are given by:

$$NMB= \frac{\sum_{i} P_{i}-O_{i}}{\sum_{i} O_{i}}\times100$$

$$NME= \frac{\sum_{i} \left| P_{i}-O_{i} \right|}{\sum_{i} O_{i}}\times100$$

where, for monitor location $i$, $P_{i}$ are the model predictions and $O_{i}$ are the observations of annual-average pollutant concentrations.

To provide context for the model-measurement comparison results, we reported model criteria published by Emery *et al.* [55]. Performance criteria were provided as a general reference point, not as “pass/fail” criteria. The criteria are intended for evaluating PM_2.5_ concentrations over sub-annual lengths of time [56], or for daily average measurements within 1000 km, where there are more than 10 measurements [55]. Here, we used the criteria more broadly to identify the stronger and weaker aspects of model performance.

Model criteria for PM_2.5_, pSO_4_, and pNH_4_ concentrations, are R^2^ ≥ 0.16, NME ≤ 50%, and |NMB| ≤ 30%. For pNO_3_, model criteria are NME ≤ 115%, |NMB| ≤ 65% (with no criteria for R^2^).

For model-to-model comparisons, weighted NMB and NME are given by:

$${NMB}_{weighted}= \frac{\sum_{i} {(GI}_{i}-M_{i})\times w_{i}}{\sum_{i} M_{i}\times w_{i}}\times100$$

$${NME}_{weighted}= \frac{\sum_{i} \left| {GI}_{i}-M_{i} \right|\times w_{i}}{\sum_{i} M_{i}\times w_{i}}\times100$$

Where $w_{i}$ are the weights (areas or population counts) for each grid cell$i$, $GI$ are the Global InMAP predictions, and $M$ are the predictions from the other model (GEOS-Chem or US InMAP).

**Table A. Names and descriptions of GEOS-Chem outputs used to calculate Global InMAP parameters.**

| Name(s) | Description and use in Global InMAP preprocessor |
| --- | --- |
| BENZ, TOLU, XYLE, NAP, POG1, POG2 | Anthropogenic VOCs that are SOA precursors; used to determine VOC/SOA partitioning |
| ASOA1, ASOA2, ASOA3, ASOAN | Anthropogenic SOA; used to determine VOC/SOA partitioning |
| ISOP, LIMO, MTPA, MTPO | Biogenic VOCs that are SOA precursors; used for model evaluation |
| TSOA0, TSOA1, TSOA2, TSOA3, SOAGX, SOAMG, SOAIE, SOAME, LVOCOA, ISN1OA | Biogenic SOA; used for model evaluation |
| NO, NO2 | Components of NO_x_; used to determine NO_x_ /pNO_3_ partitioning |
| NIT, NITs | Components of pNO_3_; used to determine NO_x_ /pNO_3_ partitioning |
| SO2 | Gaseous SO_2_ and sulfate; used to determine SO_x_ /pSO_4_ partitioning |
| SO4, SO4s, DMS | Particulate SO_4_; used to determine SO_x_ /pSO_4_ partitioning |
| NH3 | Ammonia; used to determine NH_3_/pNH_4_ partitioning |
| NH4 | Particulate Ammonium; used to determine NH_3_/pNH_4_ partitioning |
| 1.33×(NH4 + NIT + SO4) + BCPI + BCPO + 1.4×(POA1 + POA2) + 2.1×(OPOA1 + OPOA2) + 1.16×(TSOA1 + TSOA2 + TSOA3 + ASOAN + ASOA1 + ASOA2 + ASOA3 + SOAGX + INDIOL + SOAMG + SOAIE + SOAME + LVOCOA + ISN1OA) + DST1 + 0.38×DST2 + 1.86×SALA | Total PM_2.5_ concentration in the baseline simulation; used for model evaluation. 1.33, 1.16 and 1.86 factors correspond to aerosol water content; 2.1 is the global mean organic matter/organic carbon content, and 0.38 corresponds to the fraction of the DST2 tracer that is in the PM_2.5_ size fraction. |
| Z0M | Momentum roughness length |
| U, V, OMEGA | Wind fields; used to determine advection and mixing coefficients |
| PBLH | Planetary boundary layer height; used to determine mixing coefficients |
| HFLUX | Surface heat flux; used to determine mixing and dry deposition |
| USTAR | Friction velocity; used to determine mixing and dry deposition |
| T | Temperature; used to calculate chemical reaction rates and plume rise |
| PS, P | Base state pressure plus perturbation pressure; used to calculate  chemical reaction rates and plume rise |
| OH, H2O2 | Hydroxyl radical and hydrogen peroxide concentrations; used to calculate chemical reaction rates |
| FRSNO | Fraction of land covered by snow; used to calculate dry deposition |
| PFLCU, PFLLSAN | Mixing ratio of rain; used to calculate wet deposition |
| CLOUD | Fraction of grid cell covered by clouds; used to calculate wet deposition |
| QL | Cloud mixing ratio; used to calculate aqueous-phase chemical reaction rates |
| AIRDEN | Inverse air density; used to calculate mixing and to convert between  mixing ratio and mass concentration |
| PARDF, PARDR | Downward shortwave and longwave radiative flux at ground level; used to calculate dry deposition |

**Table B.** **Measurement data sources for 2016 used in evaluating Global InMAP and GEOS-Chem annual-average predictions of pollutant concentrations.** The World Health Organization data includes data from other regulatory sources and monitoring networks globally.

| Region | Data | Source |
| --- | --- | --- |
| Global | PM_2.5_ | World Health Organization |
| Europe | PM_2.5_, pNO_3_, pSO_4_, pNH_4_ | European Environment Agency |
| Canada | PM_2.5_ | National Air Pollution Surveillance Program |
| United States of America | PM_2.5_, pNO_3_, pSO_4_, pNH_4_ | Environmental Protection Agency |
| India | PM_2.5_ | Central Pollution Control Board |
| Australia | PM_2.5_ | Australian Government State of the Environment |
| East Asia | PM_2.5_, pNO_3_, pSO_4_, pNH_4_ | Acid Deposition Monitoring Network In East Asia (EANET) |

**Table C.** **Global InMAP and GEOS-Chem performance metrics for total PM_2.5_ concentrations globally, speciated PM_2.5_ concentrations globally, and total PM_2.5_ concentrations regionally.** Bold values do not meet the performance criteria (see Text A in S1 Appendix). NMB: normalized mean bias (%); NME: normalized mean error (%). Pop. wtd.: population-weighted metrics.

|  | | Global InMAP | | | | GEOS-Chem | | | | |  |
| --- | --- | --- | --- | --- | --- | --- | --- | --- | --- | --- | --- |
|  | NMB (%) | | NME (%) | R^2^ | | NMB (%) | NME (%) | | R^2^ | |  |
| Total PM_2.5_ | **-60** | | **62** | 0.33 | | **-37** | 41 | | 0.55 | |  |
| - *pop. wtd.* | -58 | | 62 | 0.57 | | -37 | 40 | | 0.75 | |  |
| pSO_4_ | 34 | | **66** | 0.29 | | 10 | 37 | | 0.53 | |  |
| pNO_3_ | -21 | | 48 | 0.46 | | -2 | 60 | | 0.34 | |  |
| pNH_4_ | 2 | | **55** | 0.25 | | **49** | **75** | | 0.42 | |  |
| Africa | **-43** | | **52** | 0.24 | | **-42** | 50 | | 0.47 | |  |
| - *pop. wtd.* | -45 | | 56 | 0.31 | | -46 | 51 | | 0.82 | |  |
| East Asia | **-52** | | **57** | 0.16 | | -28 | 32 | | 0.46 | |  |
| - *pop. wtd.* | -51 | | 55 | 0.68 | | -28 | 31 | | 0.81 | |  |
| South Asia | **-78** | | **79** | **0.07** | | **-65** | **66** | | 0.21 | |  |
| - *pop. wtd.* | -74 | | 75 | 0.26 | | -66 | 66 | | 0.68 | |  |
| Europe | **-64** | | **65** | 0.30 | | **-35** | 38 | | 0.24 | |  |
| - *pop. wtd.* | -65 | | 65 | 0.59 | | -29 | 34 | | 0.85 | |  |
| North & Central America | **-45** | | **50** | 0.92 | | -29 | 35 | | **0.08** | |  |
| - *pop. wtd.* | -55 | | 58 | 0.45 | | -34 | 41 | | 0.91 | |  |
| Oceania | **-49** | | 49 | 0.82 | | **-49** | 49 | | 0.68 | |  |
| - *pop. wtd.* | -53 | | 53 | 0.89 | | -58 | 58 | | 0.87 | |  |
| South America | **-76** | | **80** | **0.05** | | **-79** | **79** | | **0.05** | |  |
| - *pop. wtd.* | -70 | | 78 | 0.13 | | -73 | 73 | | 0.87 | |  |
|  | |  |  |  | |  |  | |  | |  |
|  | |  |  |  | |  |  | |  | |  |
|  | |  |  |  |  | | |  | |  | |

**Table D.** **Regional, population-weighted total concentration predictions**. Population-weighted concentrations for different PM_2.5_ species as predicted by Global InMAP and GEOS-Chem across different regions. (See Table F in S1 Appendix for the region extents).

| Region | Global InMAP pop-wtd. concentrations (μg m^-3^) | | | | | GEOS-Chem pop-wtd. concentrations (μg m^-3^) | | | | |
| --- | --- | --- | --- | --- | --- | --- | --- | --- | --- | --- |
|  | Primary PM_2.5_ | pSO_4_ | pNO_3_ | pNH_4_ | SOA | Primary PM_2.5_ | pSO_4_ | pNO_3_ | pNH_4_ | SOA |
| Global | 5.26 | 4.79 | 1.55 | 1.12 | 0.51 | 9.31 | 3.03 | 1.85 | 1.52 | 0.75 |
| South Asia | 5.26 | 5.62 | 1.17 | 1.08 | 0.34 | 9.48 | 4.49 | 1.26 | 1.87 | 0.75 |
| East Asia | 4.03 | 8.01 | 3.54 | 2.03 | 1.01 | 10.88 | 4.31 | 4.92 | 2.83 | 0.82 |
| Africa | 9.92 | 2.74 | 0.32 | 0.44 | 0.53 | 16.96 | 1.51 | 0.06 | 0.47 | 0.87 |
| Europe | 2.81 | 2.54 | 0.93 | 0.52 | 0.20 | 4.51 | 1.62 | 1.59 | 0.96 | 0.25 |
| South America | 1.25 | 1.91 | 0.65 | 0.89 | 0.79 | 1.42 | 1.26 | 0.07 | 0.39 | 0.76 |
| North America | 0.87 | 2.81 | 0.57 | 0.59 | 0.67 | 1.37 | 1.46 | 0.48 | 0.59 | 0.60 |
| Oceania | 3.40 | 4.09 | 0.95 | 1.47 | 1.34 | 2.86 | 3.44 | 3.44 | 1.25 | 0.44 |

**Table E.** **Regional model performance for predicted changes in pollutant concentrations.** Area- and population-weighted normalized mean bias (NMB) and error (NME) for Global InMAP predicted changes in concentrations against changes in concentrations from GEOS-Chem, arising from scenarios of changes in emissions, for the 3 most populated regions, South Asia, East Asia, and Africa. Positive bias indicates that Global InMAP has higher average concentration changes than the other model.

| Scenario | Region | Weighting | NME (%) | NMB (%) |
| --- | --- | --- | --- | --- |
| NH_3_ increase from agricultural soils | Global | area-wtd. | 118.2 | 58.7 |
|  |  | population-wtd. | 81.8 | 58.7 |
|  | South Asia | area-wtd. | 75.2 | 41.1 |
|  |  | population-wtd. | 44.4 | 4.7 |
|  | East Asia | area-wtd. | 111.5 | 49.1 |
|  |  | population-wtd. | 78.9 | 16.4 |
|  | Africa | area-wtd. | 204.9 | 189.0 |
|  |  | population-wtd. | 181.2 | 173.1 |
| NO_x_ increase from road transportation | Global | area-wtd. | 180.7 | 96.2 |
|  |  | population-wtd. | 106.7 | 48.9 |
|  | South Asia | area-wtd. | 148.3 | 86.2 |
|  |  | population-wtd. | 59.4 | -15.2 |
|  | East Asia | area-wtd. | 158.5 | 138.5 |
|  |  | population-wtd. | 127.6 | 110.5 |
|  | Africa | area-wtd. | 277.5 | 207.4 |
|  |  | population-wtd. | 156.4 | 89.6 |
| SO_x_ increase from power generation | Global | area-wtd. | 181.3 | 120.7 |
|  |  | population-wtd. | 275.4 | 216.9 |
|  | South Asia | area-wtd. | 84.7 | 14.2 |
|  |  | population-wtd. | 56.7 | 23.9 |
|  | East Asia | area-wtd. | 202.1 | 140.1 |
|  |  | population-wtd. | 381.9 | 371.0 |
|  | Africa | area-wtd. | 225.2 | 205.0 |
|  |  | population-wtd. | 262.4 | 254.4 |

**Table F. Region definitions.** Longitude and latitude extents for the regions and cities referenced in this study.

| Region | Longitude | Latitude |
| --- | --- | --- |
| East Asia | (95, 145) | (5, 58) |
| *- Tokyo* | (138, 142) | (35, 38) |
| South Asia | (50, 95) | (5, 33) |
| Africa | (-18, 58) | (-37, 40) |
| *- Cairo* | (29, 33) | (29, 32) |
| Europe | (-14, 49) | (33, 71) |
| North America | (-135, -58) | (9, 53) |
| Oceania | (95, 189) | (-48, 12) |
| South America | (-87, -31) | (-59, 14) |
| *- São Paulo* | (-45, -49) | (-22, -25) |
