# Supplementary figures and images for: Global, high-resolution, reduced-complexity air quality modeling for PM2.5 using InMAP (Intervention Model for Air Pollution)
Source: PLoS One. 2022 May 25;17(5):e0268714. doi: 10.1371/journal.pone.0268714 (PMC9132322; doi:10.1371/journal.pone.0268714)

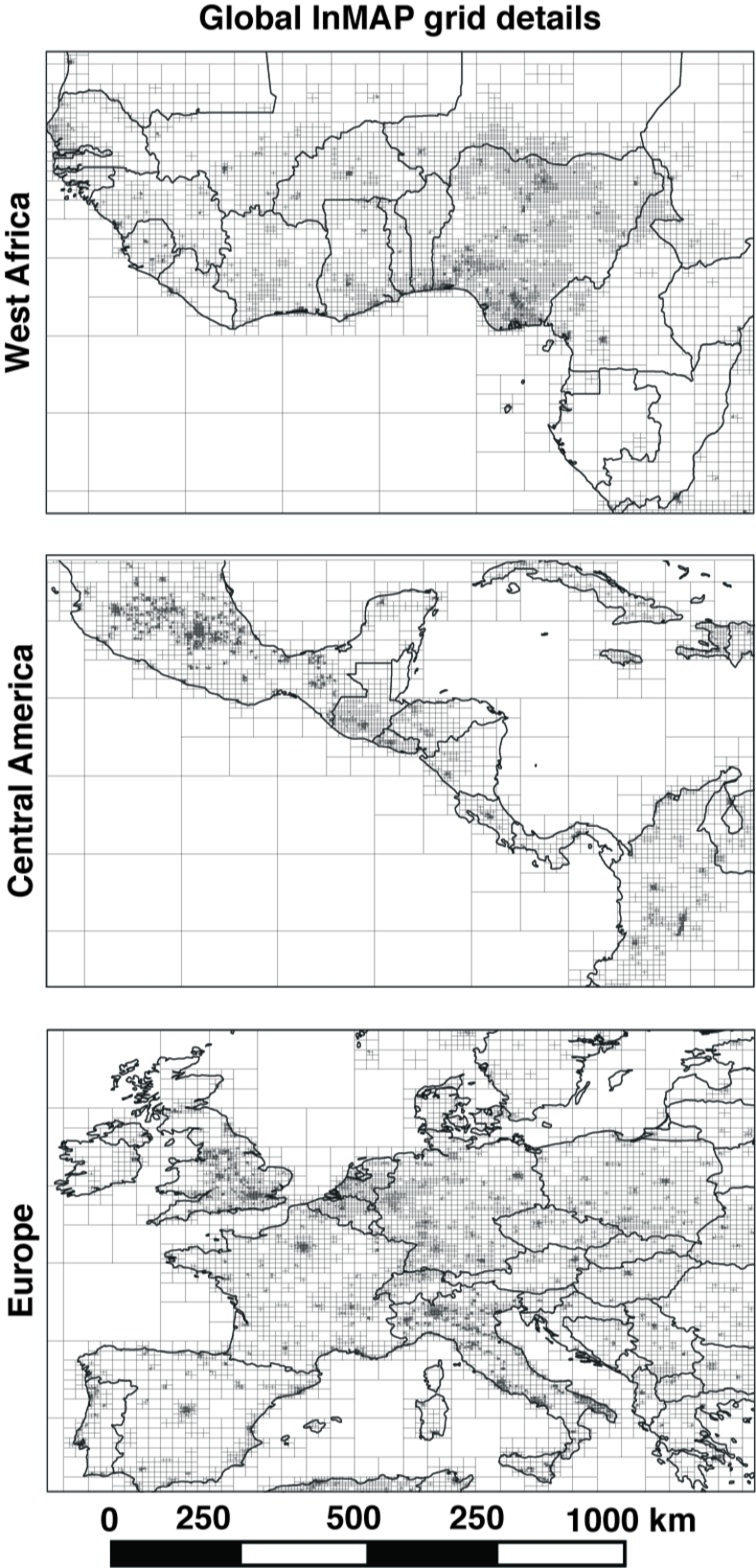

Supplement: S1 Fig — Detail of the Global InMAP horizontal computational grid over West Africa, Central America, and Europe for illustration. Grid cells are as small as 0.04° × 0.03° (~4 km length) in areas with a higher population such as Lagos in Nigeria, San Salvador in El Salvador, and London in the United Kingdom. Grid cells are as large as 5° × 4° (~500 km length) in places with a lower population, such as across the Atlantic Ocean. (TIFF) [file pone.0268714.s002.tiff]

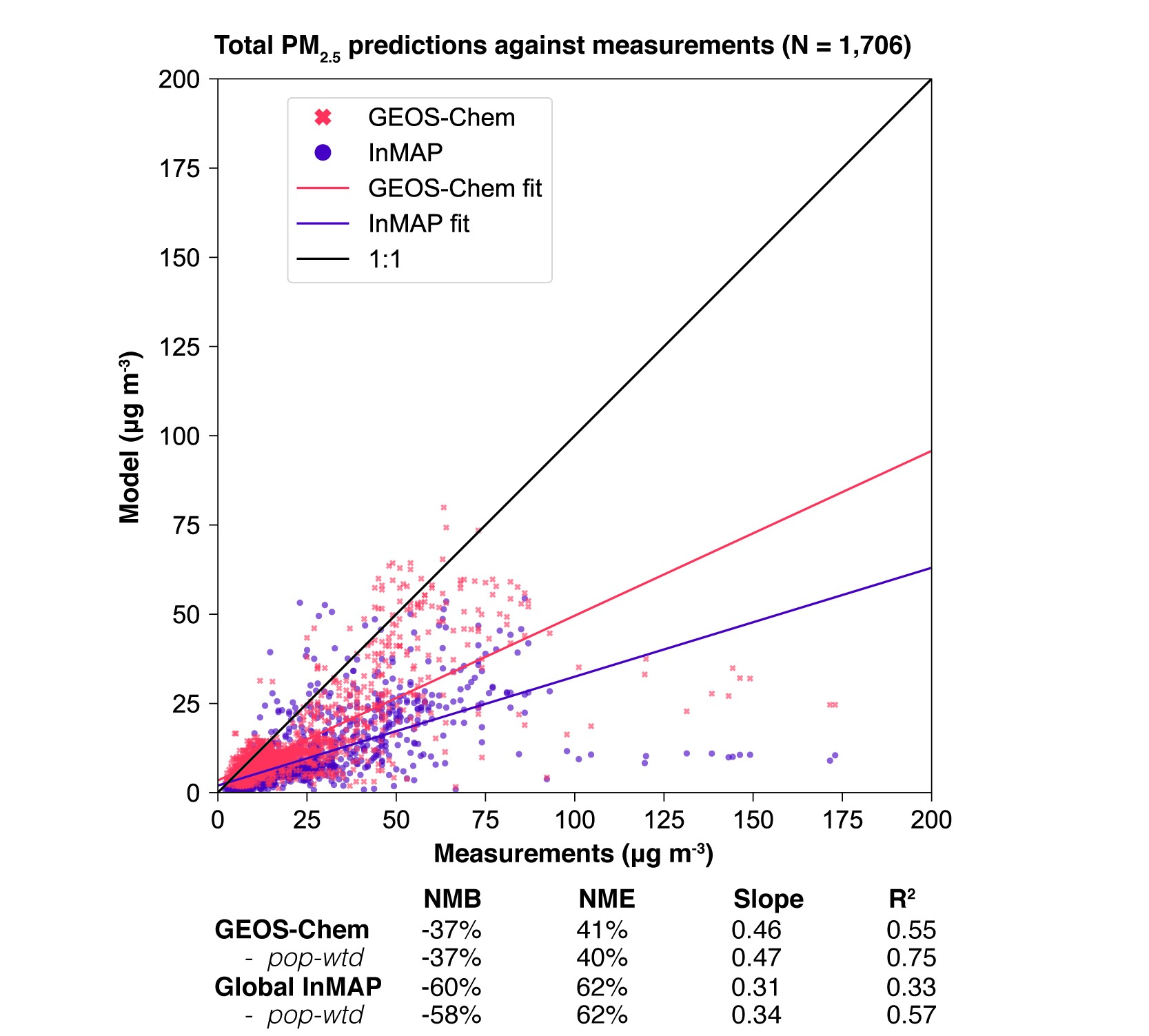

Supplement: S2 Fig — InMAP and GEOS-Chem annual-average primary PM2.5 concentrations against measurements, including outliers (above 100 μg m-3). Pop-wtd: Population-weighted metrics. (TIFF) [file pone.0268714.s003.tiff]

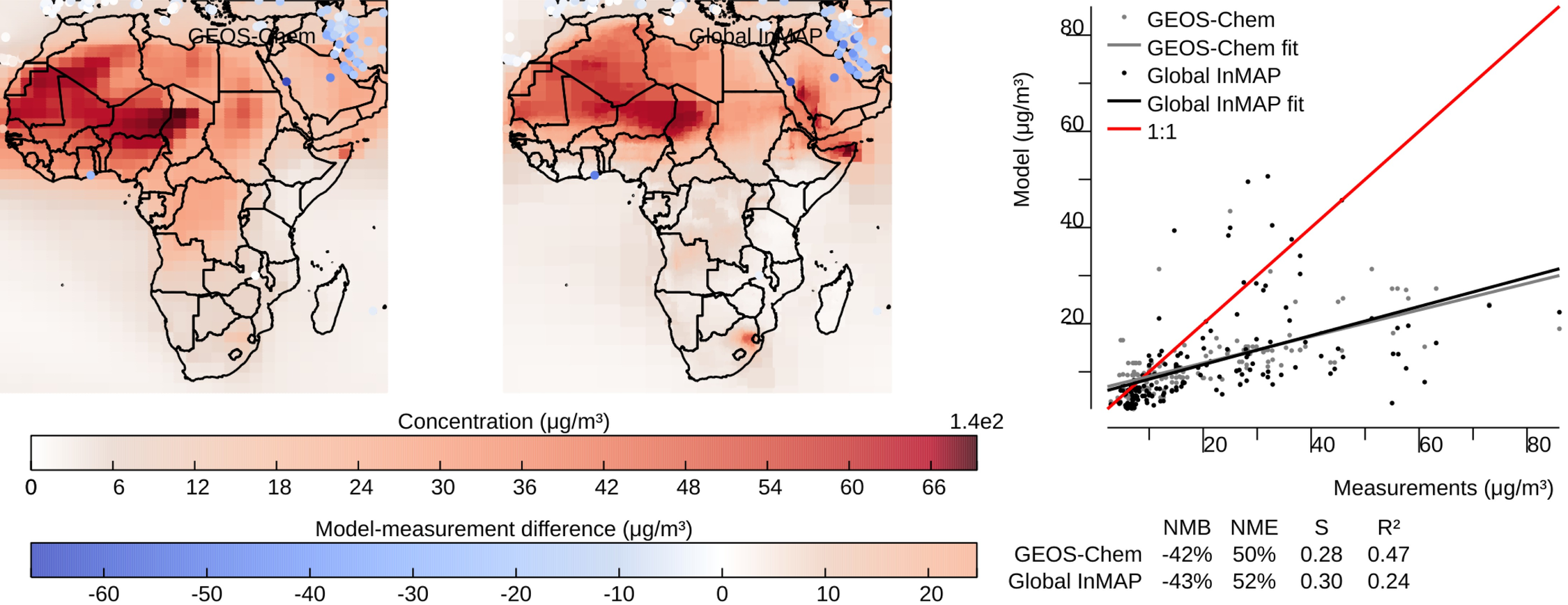

Supplement: S3 Fig — Performance of Global InMAP and GEOS-Chem simulations against total annual-average PM2.5 measurements for Africa. Dots on each map show measurement site locations, whose color corresponds to the model-measurement difference in PM2.5 concentrations. (TIFF) [file pone.0268714.s004.tiff]

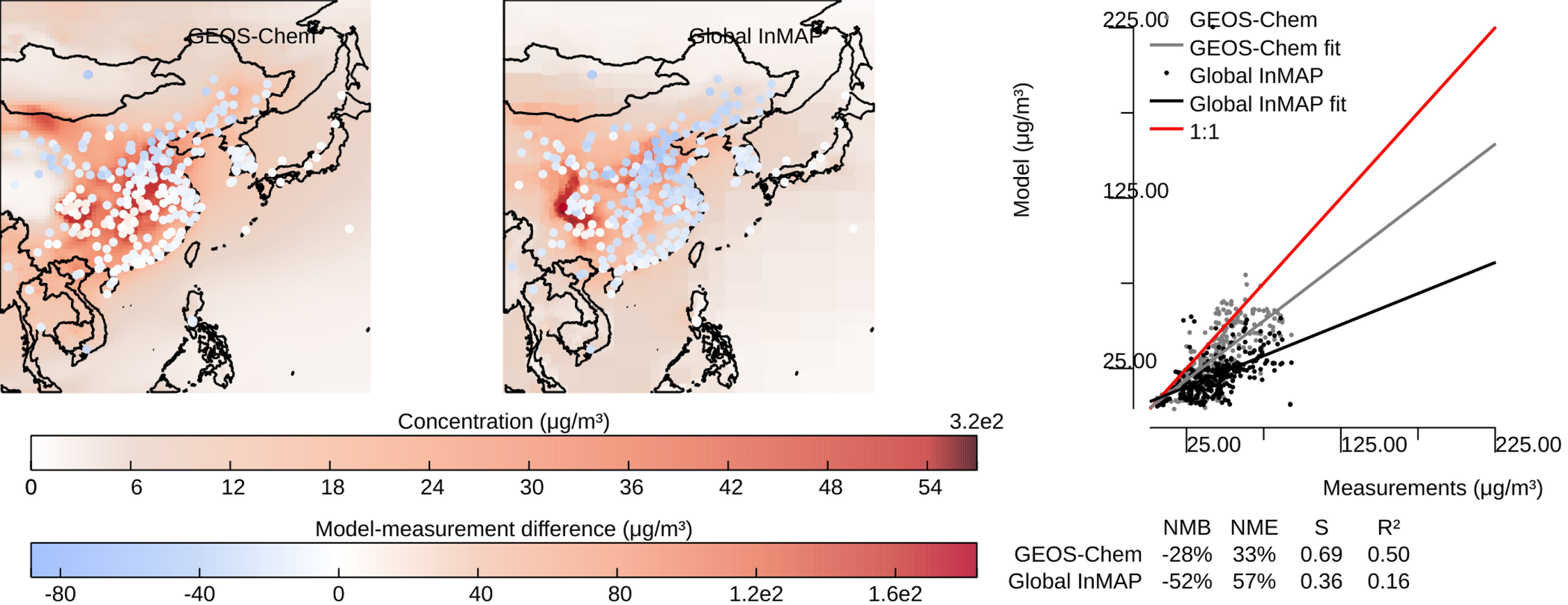

Supplement: S4 Fig — Performance of Global InMAP and GEOS-Chem simulations against total annual-average PM2.5 measurements for East Asia. Dots on each map show measurement site locations, whose color corresponds to the model-measurement difference in PM2.5 concentrations. (TIFF) [file pone.0268714.s005.tiff]

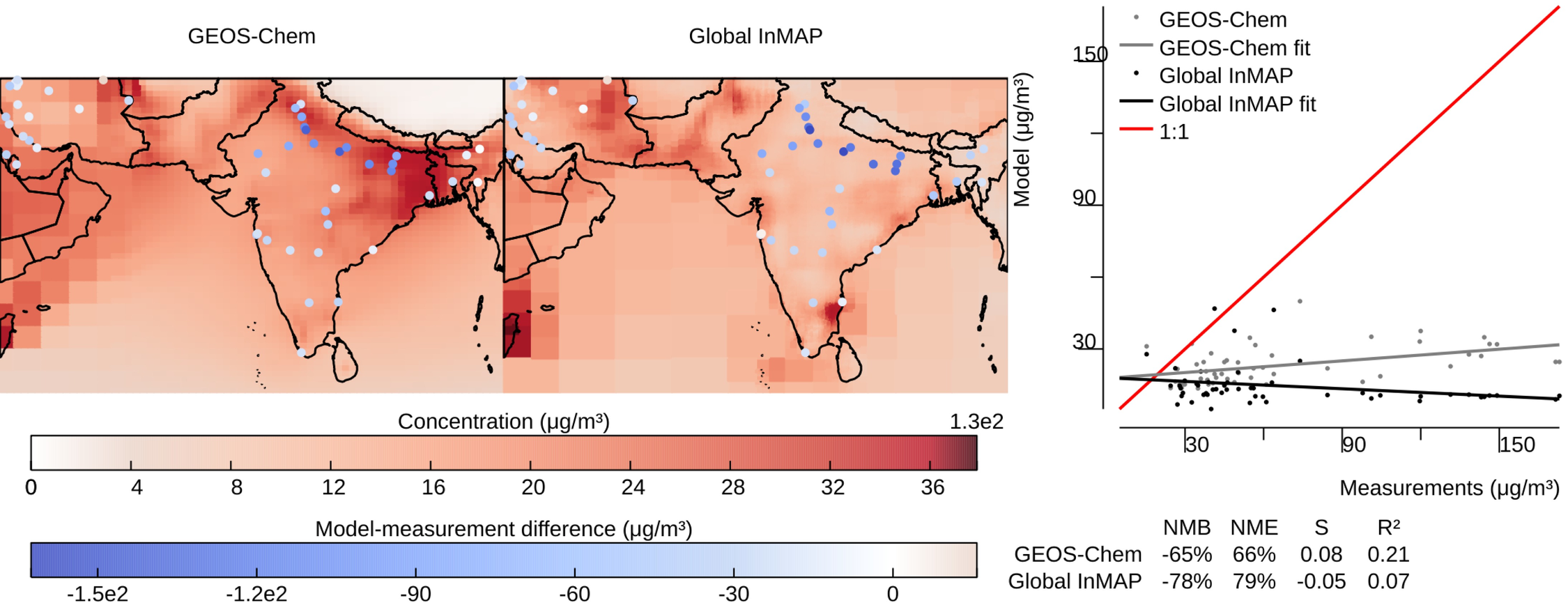

Supplement: S5 Fig — Performance of Global InMAP and GEOS-Chem simulations against total annual-average PM2.5 measurements for South Asia. Dots on each map show measurement site locations, whose color corresponds to the model-measurement difference in PM2.5 concentrations. (TIFF) [file pone.0268714.s006.tiff]

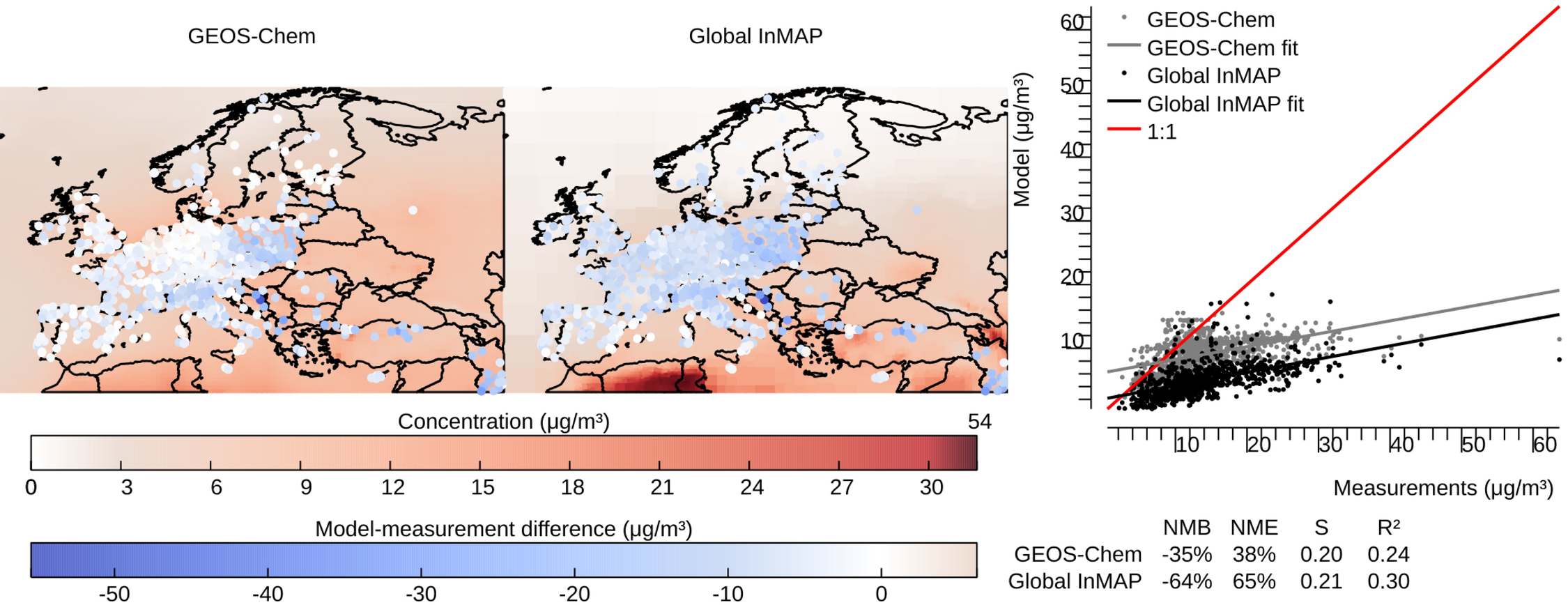

Supplement: S6 Fig — Performance of Global InMAP and GEOS-Chem simulations against total annual-average PM2.5 measurements for Europe. Dots on each map show measurement site locations, whose color corresponds to the model-measurement difference in PM2.5 concentrations. (TIFF) [file pone.0268714.s007.tiff]

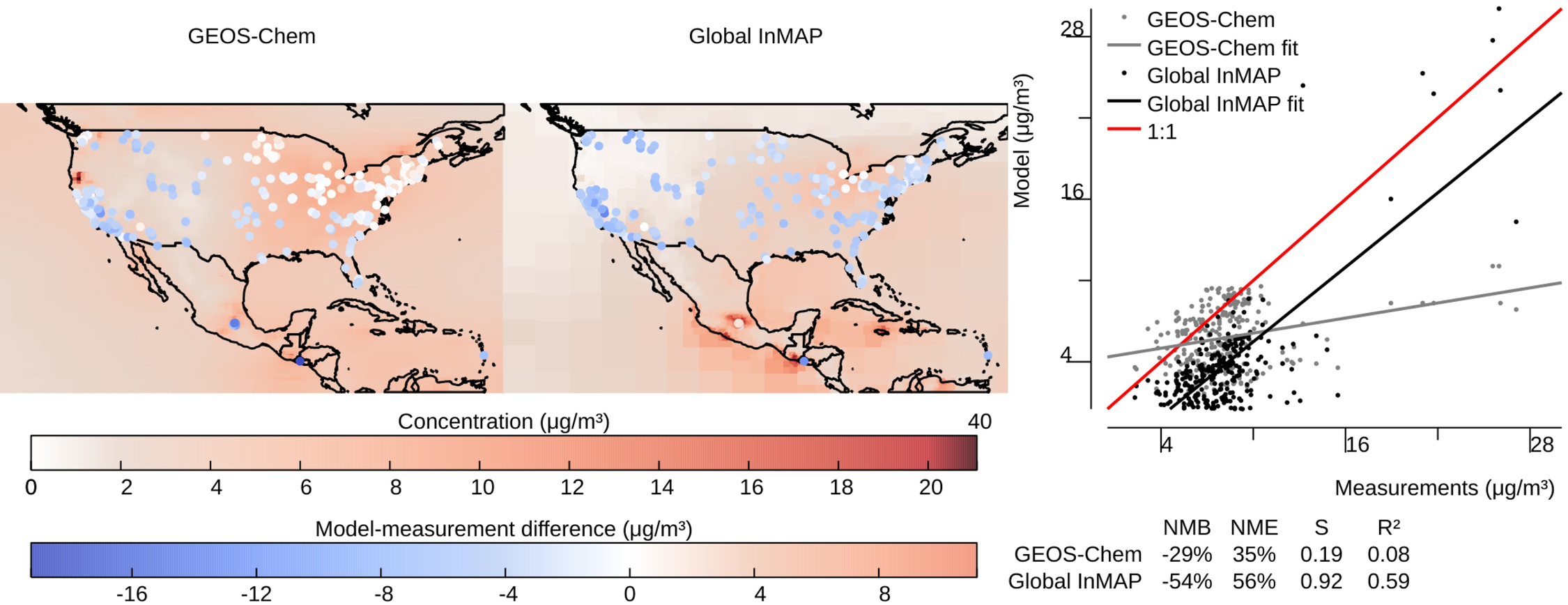

Supplement: S7 Fig — Performance of Global InMAP and GEOS-Chem simulations against total annual-average PM2.5 measurements for North and Central America. Dots on each map show measurement site locations, whose color corresponds to the model-measurement difference in PM2.5 concentrations. (TIFF) [file pone.0268714.s008.tiff]

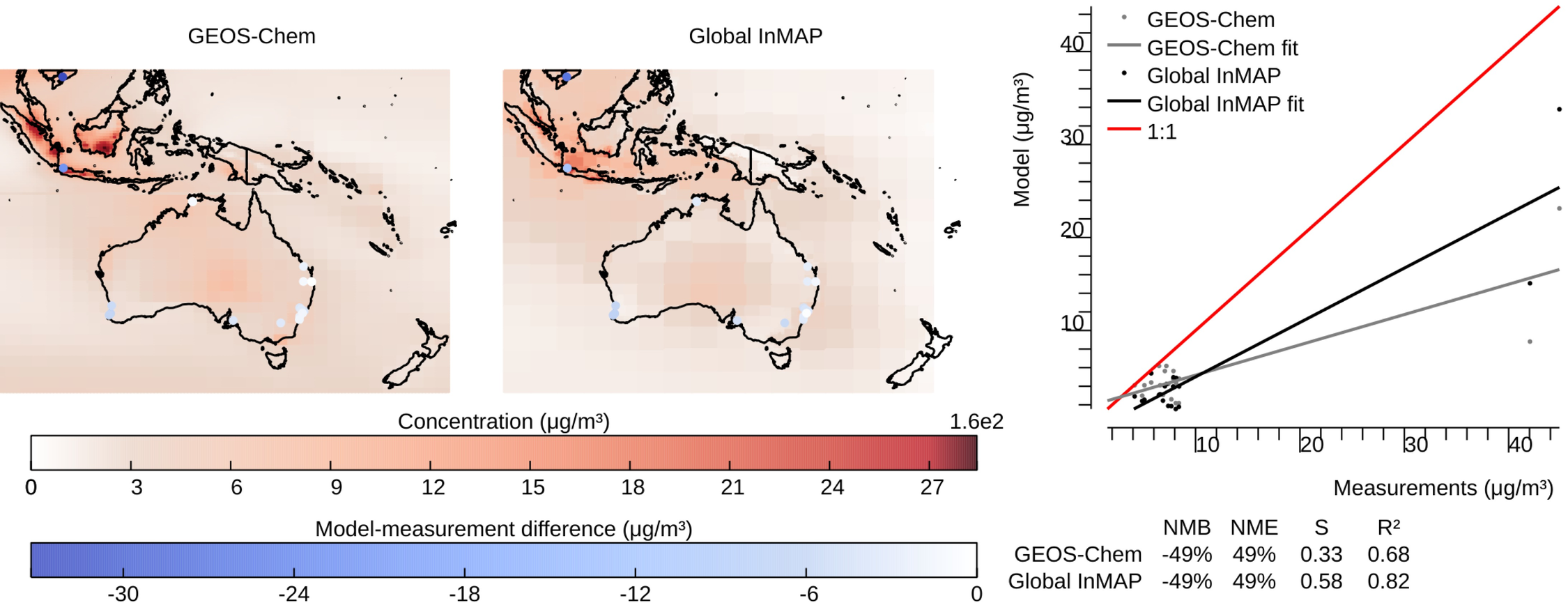

Supplement: S8 Fig — Performance of Global InMAP and GEOS-Chem simulations against total annual-average PM2.5 measurements for Oceania. Dots on each map show measurement site locations, whose color corresponds to the model-measurement difference in PM2.5 concentrations. (TIFF) [file pone.0268714.s009.tiff]

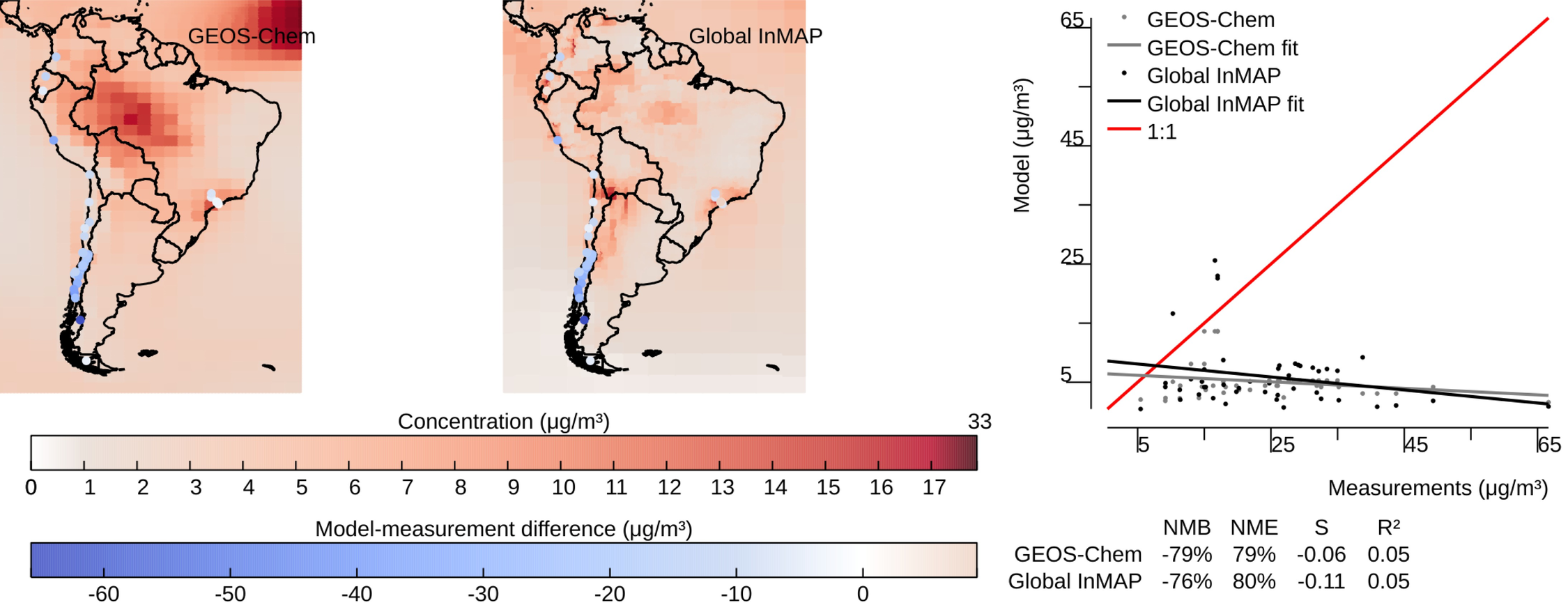

Supplement: S9 Fig — Performance of Global InMAP and GEOS-Chem simulations against total annual-average PM2.5 measurements for South America. Dots on each map show measurement site locations, whose color corresponds to the model-measurement difference in PM2.5 concentrations. (TIFF) [file pone.0268714.s010.tiff]

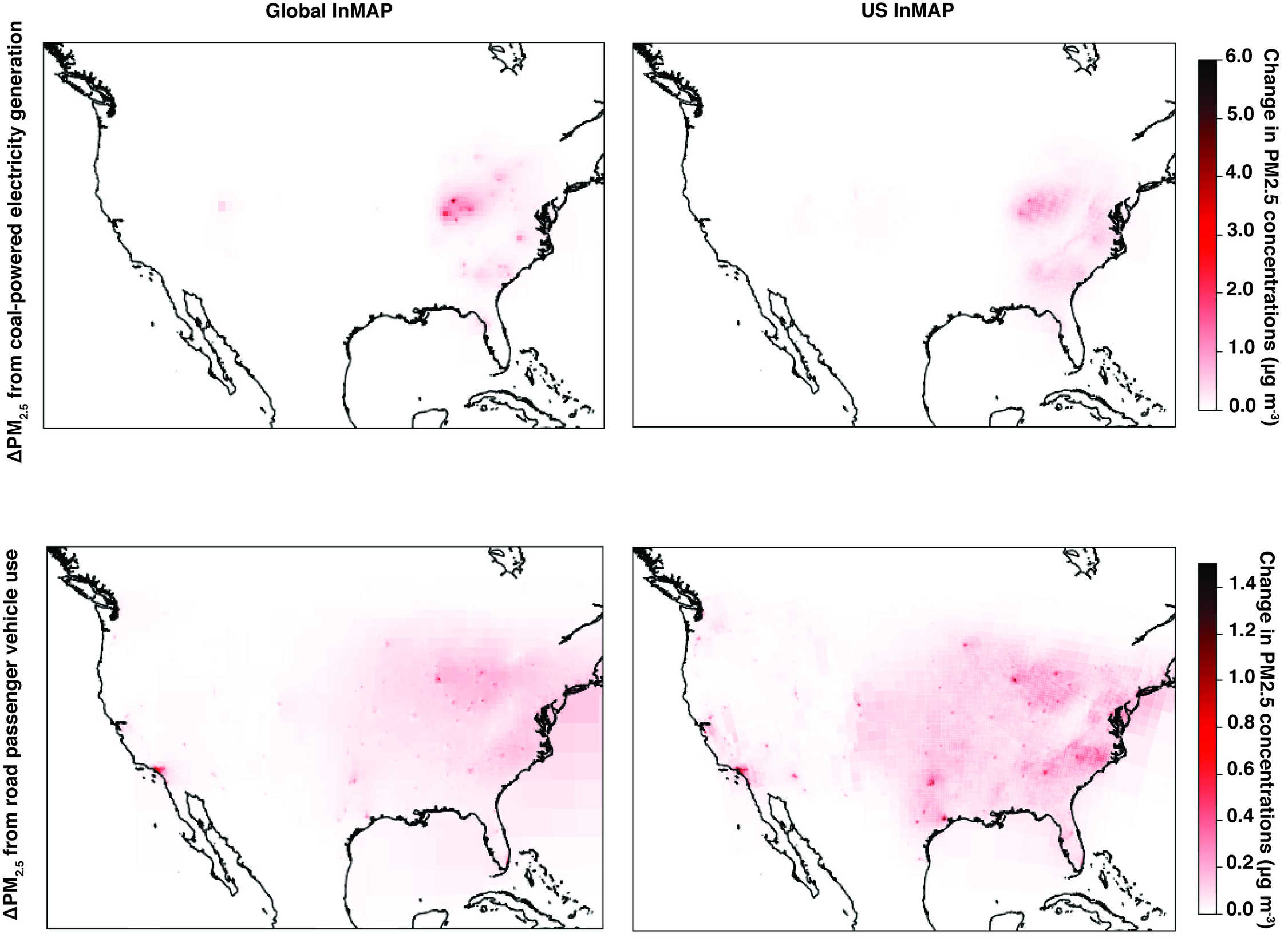

Supplement: S10 Fig — Changes in Total PM2.5 concentrations from road vehicle emissions and from power generation emissions as predicted by Global InMAP (which has GEOS-Chem preprocessor inputs) alongside US InMAP (which has WRF-Chem preprocessor inputs). (TIFF) [file pone.0268714.s011.tiff]
